# Uniqueness of Solutions to Nonlinear Schrödinger Equations from their Zeros

**DOI:** 10.1007/s40818-022-00138-1

**Published:** 2022-09-14

**Authors:** Christoph Kehle, João P. G. Ramos

**Affiliations:** 1 Institute for Advanced Study, School of Mathematics, 1 Einstein Drive, Princeton, NJ USA; 2 ETH Zürich, Institute for Theoretical Studies, Clausiusstrasse 47, 8092 Zurich, Switzerland; 3 ETH Zürich, Department of Mathematics, Rämisstrasse 101, 8092 Zurich, Switzerland

## Abstract

We show novel types of uniqueness and rigidity results for Schrödinger equations in either the nonlinear case or in the presence of a complex-valued potential. As our main result we obtain that the trivial solution $u=0$ is the only solution for which the assumptions $u(t=0)\vert _{D}=0, u(t=T)\vert _{D}=0$ hold, where $D\subset \mathbb {R}^d$ are certain subsets of codimension one. In particular, *D* is *discrete* for dimension $d=1$. Our main theorem can be seen as a nonlinear analogue of discrete Fourier uniqueness pairs such as the celebrated Radchenko–Viazovska formula in [21], and the uniqueness result of the second author and M. Sousa for powers of integers [22]. As an additional application, we deduce rigidity results for solutions to some semilinear elliptic equations from their zeros.

## Introduction

Let $f \in L^2(\mathbb {R}).$ The problem of determining necessary and sufficient conditions on sets *A*, *B* so that $f|_A = {\widehat{f}}|_B = 0$ implies $f \equiv 0$ has been extensively studied in connection with the celebrated Heisenberg uncertainty principle. If a pair of sets (*A*, *B*) possesses the property stated above, possibly under additional assumptions on the functions *f*, we call it a *Fourier uniqueness pair*.

For instance, $(A,B) = (\mathbb {R}\setminus (-1/2,1/2), \mathbb {Z})$ is seen, due to the Shannon–Whittaker interpolation formula for band-limited functions, to be a Fourier uniqueness pair. In the celebrated work [21], Radchenko and Viazovska recently proved that, restricted to the class of Schwartz functions, $(A,B) = (\sqrt{n},\sqrt{n})_{n\in {\mathbb {N}}}$ is a Fourier uniqueness pair. After the Radchenko–Viazovska breakthrough, several other results came about giving necessary and sufficient conditions on the pair (*A*, *B*) so that the Fourier uniqueness property holds, for which we refer the reader to [15, 23, 24] and the references therein.

While the Fourier transform is a rather distinguished and highly symmetric operator, it is now a natural problem to explore the validity of such uniqueness results for more general operators which are known to enjoy similar uncertainty properties. Indeed, in the recent work [7] by F. Gonçalves and the second author, the first step connecting Fourier uniqueness pairs to other operators has been taken. There, the authors consider the *free Schrödinger equation*

1.1 $$\begin{aligned} {\left\{ \begin{array}{ll} i \partial _t u + \Delta u = 0 &{} \text { in } \mathbb {R}\times \mathbb {R}; \\ u(x,0) = u_0(x) &{} \text { on } \mathbb {R}. \\ \end{array}\right. } \end{aligned}$$

Among other properties, the authors prove that, for certain discrete subsets of the $(x,t)-$plane, if a solution *u* vanishes at the points of that set, and $u_0$ belongs to a fast-decaying class of functions, then $u \equiv 0.$

The result relies heavily on the explicit representation of the free evolution of (1.1) given by $u = e^{it\Delta } u_0 = \frac{1}{ \sqrt{4t \pi i} } e^{-|x|^2/4it} *u_0$ which allows to resemble several aspects of the previously mentioned rigidity results for the Fourier transform. An important question immediately raised is whether such uniqueness results extend for the evolution in the presence of a given *potential* or for *nonlinear interactions*. Indeed, already in the linear case of the Schrödinger equation with a (possibly complex-valued) potential

1.2 $$\begin{aligned} {\left\{ \begin{array}{ll} i \partial _t u + \Delta u + V u= 0 &{} \text { in } \mathbb {R}\times [0,T]; \\ u(x,0) = u_0(x) &{} \text { on } \mathbb {R}, \\ \end{array}\right. } \end{aligned}$$

the techniques used by Gonçalves and the second author [7], which mainly rely on relating the evolution of the free Schrödinger equation to suitable Fourier transforms, completely fall apart, and it seems that new techniques are needed then.

The main purpose of this work is to provide first examples of *discrete* uniqueness sets for the Schrödinger equation, either in the case of a potential or in the nonlinear case. Before we introduce our main results in Section 2, however, we summarize some previous progress on the question of unique continuation for the Schrödinger equation.

Indeed, our starting point will be a result on unique continuation for the Schrödinger equation by Kenig, Ponce and Vega [14], where the authors proved the following. Let $u_1,u_2 \in C([0,T]; H^s(\mathbb {R}^d))),$ $\, s \ge \max \{d/2+,2\},$ be solutions to

1.3 $$\begin{aligned} i \partial _t u + \Delta u + F(u,{\overline{u}}) = 0 \text { in } \mathbb {R}^d\times [0,T], \end{aligned}$$

where $F \in C^{\lfloor s \rfloor + 1} (\mathbb {C}^2 :\mathbb {C})$ satisfies $|\nabla F(u,{\overline{u}})| \lesssim (|u|^{p_1 - 1} + |u|^{p_2 - 1}), p_1, p_2 >1.$ If

$$\begin{aligned} u_1(x,0) = u_2(x,0), \, \, u_1(x,T) = u_2(x,T), \, \, \forall x \not \in \Gamma + y_0, \end{aligned}$$

where $\Gamma$ is a convex cone contained in a half space, then $u_1 \equiv u_2.$ One of the key steps in their proof is a lemma—which we shall use in this paper; see Lemma 5.1 for more details—which roughly states that exponential decay is preserved for times $t \in (0,T),$ as long as we have exponential decay of the solution at two times $t=0,T.$

After the Kenig–Ponce–Vega breakthrough, several other advances in the problem of determining sharper conditions on $u_1-u_2$ for uniqueness have been made, such as [16], the recent survey [17] for non-local operators and the references therein. In particular, in the series of papers [3–5], Escauriaza–Kenig–Ponce–Vega obtained a *sharp form* of Hardy’s uncertainty principle for the Schrödinger evolution: suppose $u \in C([0,T];L^2(\mathbb {R}^d))$ is a solution to (1.2), and suppose additionally that there are two constants $\alpha ,\beta > 0$ with $\frac{\alpha \beta }{4T} < 1$ such that

1.4 $$\begin{aligned} \Vert e^{|x|^2/\beta ^2} u(0)\Vert _2 + \Vert e^{|x|^2/\alpha ^2} u(T)\Vert _2 <+\infty . \end{aligned}$$

If the potential *V* satisfies $\lim _{R \rightarrow \infty } \Vert V\Vert _{L^1([0,T];L^{\infty }(\mathbb {R}^d \setminus B_R))} = 0,$ then one has $u \equiv 0.$ They also proved that the conditions on $\alpha ,\beta$ are *sharp*, as in the case of $\alpha \beta = 4T$ there is a potential satisfying the condition above and a non-zero solution to the Schrödinger equation (1.2) satisfying (1.4).

As a consequence of their results for the potential case (1.2), they obtain that if two solutions $u_1,u_2 \in C([0,T];H^k(\mathbb {R}^d)), k > d/2,$ of (1.3), with $F \in C^k, \, F(0) = \partial _u F(0) = \partial _{{\overline{u}}} F(0) = 0,$ satisfy

$$\begin{aligned} e^{|x|^2/\beta ^2} (u_1(0) - u_2(0)), e^{|x|^2/\alpha ^2} (u_1(T) - u_2(T)) \in L^2, \end{aligned}$$

where $$\alpha \beta < 4T,$$ then $$u_1 \equiv u_2.$$

## Main Results

As highlighted before, the results above deal with decay properties of solutions to Schrödinger equations on *large* spatial sets, in particular sets with zero codimension. Our main results are concerned with knowledge of the solution on *small* spatial sets with codimension one. In particular, in the one-dimensional case, we obtain rigidity already from zeros (or decay) of the solution imposed on a *discrete* set.

We first consider the one dimensional case, where we show a robust rigidity result for the Schrödinger equation in the presence of a complex potential (Theorem 1) and for general nonlinearities (Theorem 1). The theorem, however, only applies in dimension $d=1$ and we have to assume rapidly accumulating zeros (or decay) at infinity. This is the content of Section 2.1. Then, in Section 2.2, we state our main rigidity results Theorem 2 ($d=1$) and Theorem 3 ($d\ge 2$) for the cubic NLS assuming only a sparser set of zeros. In Section 2.3 we present a novel rigidity result (Corollary 2) for certain semilinear elliptic PDEs as a corollary from the main results from Section 2.2. Finally, in Section 2.4 we give an outlook how to extend the main theorems of Section 2.2 to more general power nonlinearities for the Schrödinger equation.

### Main Results for Rapidly Accumulating Zeros for Complex Potential and General Nonlinearities in $d=1$

In dimension $d=1,$ we are able to prove, through basic estimates on the linear unitary group $\{e^{it\partial _x^2}\}_{t \in \mathbb {R}}$ and some cleaner estimates, the following result on uniqueness under quite weak regularity conditions.

#### Theorem 1

Let $u \in C([0,T] :L^2(\mathbb {R}))$ be a strong solution of the initial value problem associated with the Schrödinger equation with complex-valued potential:

2.1 $$\begin{aligned} {\left\{ \begin{array}{ll} i \partial _t u = - \partial _x^2 u + V u &{} \text { in } \mathbb {R}\times [0,T], \\ u(x,0) = u_0(x) &{} \text { on } \mathbb {R}, \\ \end{array}\right. } \end{aligned}$$

where we assume that the potential satisfies $V \in L^1([0,T] :L^{\infty }(\mathbb {R})) \cap L^{\infty }([0,T] :L^2((1+|x|)^2 dx))$ and $\lim _{R \rightarrow \infty } \Vert V \Vert _{L^1_{[0,T]} L^{\infty }(\mathbb {R}\setminus B_R)} = 0$.

Suppose $u_0, u(T) \in L^1((1+|x|)dx)$ and that there are constants $c_1,c_2,\delta > 0$ so that, for some $\alpha \in (0, \frac{1}{2}),$ we have

2.2 $$\begin{aligned} |u_0(\pm c_1 \log (1+n)^{\alpha })| + |u(\pm c_2 \log (1+n)^{\alpha },T)| \le n^{-\delta }, \, \forall n \ge 0. \end{aligned}$$

Then, we have $u \equiv 0.$

#### Remark 2.1

The assumptions on the solution $u_0,u(T) \in L^1( (1+|x|) dx)$ and on the potential $V \in L^1([0,T] :L^{\infty }(\mathbb {R})) \cap L^{\infty }([0,T] :L^2((1+|x|)^2 dx))$ will guarantee that $u_0, u(T) \in C^{\frac{1}{2}}(\mathbb {R})$ (see already the proof of Theorem 1 in Section 5.1). Thus, the pointwise conditions on $u_0$ and *u*(*T*) in (2.2) are well-defined.

The proof of Theorem 1, given in Section 5.1, departs from the Duhamel formula for the solution *u*. Then, we use the two dispersive estimates associated to $e^{it\partial _x^2}$ (see already Lemma 3.1) in different regimes in the Duhamel formula. Under the assumptions of Theorem 1, we will show that we subsequently obtain sub-Gaussian decay of the solution at two different times. By the results [3–5] of Escauriaza–Kenig–Ponce–Vega highlighted above, we must have that each solution with those properties is identically zero.

As a consequence of Theorem 1, we are able to prove the following corollary, which asserts that if two solutions of (1.3), which are of class $H^s, s > 1/2,$ possess some mild spatial decay and nearly coincide at many points, then they must be the same.

#### Corollary 1

Let $u,v \in C([0,T] :H^{s}(\mathbb {R}) \cap L^2(x^2 \, dx)), s > 1/2,$ be two solutions of the initial value problem associated with the Schrödinger equation with a nonlinear term:

2.3 $$\begin{aligned} {\left\{ \begin{array}{ll} i \partial _t w=- \partial _x^2 w + F(w,{\overline{w}}) &{} \text { in } \mathbb {R}\times [0,T], \\ w(x,0) = w_0(x) &{} \text { on } \mathbb {R}, \\ \end{array}\right. } \end{aligned}$$

where $$F \in C^1(\mathbb {C}^2 :\mathbb {C})$$ satisfies

2.4 $$\begin{aligned} |\nabla F(w,{\overline{w}})| \le C|w|^{p-1} \end{aligned}$$

for some $p>2$. Suppose that there are constants $c_1,c_2,\delta > 0$ so that, for some $\alpha \in (0, \frac{1}{2}),$ we have

$$\begin{aligned} |(u_0-v_0)(\pm c_1 \log (1+n)^{\alpha })| + |(u-v)(\pm c_2 \log (1+n)^{\alpha },T)| \le n^{-\delta }, \, \forall n \ge 0 \end{aligned}$$

as well as $u_0 - v_0, u(T) - v(T) \in L^1((1+|z|)dz)$. Then, we have $u \equiv v.$

Notice that Corollary 1 is much easier in the case of $u,v \in C([0,T], H^1(\mathbb {R})),$ as then the fundamental theorem of calculus gives us the sub-Gaussian estimates in a cheap way. This is, in part, the reason why that result is only stated in one dimension: for higher dimensions, the “natural" condition would be $s > d/2,$ in which case a Poincaré inequality argument—see the proof of Lemma 4.9 for more details—would also give us the result almost instantly.

### Main Results for Slowly Accumulating Zeros for Cubic NLS for $d\ge 1$

Notice that the conditions on the set where *u* is “small" in Theorem 1—though discrete—are quite dense, in the sense that, given regularity, the Poincaré inequality argument as before already gives the result by previous estimates. On the other hand, if the nodes are “sparser" (i.e. $\alpha \in (0,1)$ instead of $\alpha \in (0,\frac{1}{2})$ as in Corollary 1), then sub-Gaussian decay cannot be obtained as described above. We will address this question for the cubic NLS

2.5 $$\begin{aligned} {\left\{ \begin{array}{ll} i \partial _t u = -\Delta u - \lambda |u|^2 u &{} \text { in } [0,T] \times \mathbb {R}^d, \\ u(x,0) = u_0(x) &{} \text { on } \mathbb {R}^d, \end{array}\right. } \end{aligned}$$

for *fixed* $\lambda \in {\mathbb {C}}$. We first state our result for dimension $d=1$.

#### Theorem 2

Let $u \in C([0,T] :L^2(\mathbb {R}))$ be a strong solution to the initial value problem (2.5) for $d=1$.

Suppose additionally that $u_0, u(T) \in L^2(x^2 \, dx),$ and that the solution satisfies

2.6 $$\begin{aligned} u_0( \pm c_1 \log (1+n)^{\alpha }) = u(\pm c_2 \log (1+n)^{\alpha },T) = 0, \, \forall \, n \ge 0, \end{aligned}$$

for some $\alpha \in (0,1)$ and some $c_1,c_2 >0$. Then $u \equiv 0.$

#### Remark 2.2

By local smoothing of the Schrödinger equation (see already Lemma 4.1), the assumptions $u_0,u(T) \in L^2(x^2 dx)$ will guarantee $u_0, u(T) \in H^1(\mathbb {R})$ and are thus sufficient to make sense of the pointwise conditions on $u_0$ and *u*(*T*) in (2.6).

#### Remark 2.3

The seemingly arithmetic nature of the zero sets in the statement of Theorem 2 does not actually play a major role in its proof. As a matter of fact, the main property needed is a control on the gap between consecutive roots. That is, it would be enough to have $u_0$ and *u*(*T*) vanish at sequences of points $\{\lambda _n\}_{n \in \mathbb {Z}}$ and $\{\gamma _n\}_{n \in \mathbb {Z}}$, respectively, which satisfy

$$\begin{aligned}&\lim _{n\rightarrow \pm \infty } \lambda _n = \lim _{n\rightarrow \pm \infty } \gamma _n = \pm \infty , \\&|\lambda _{n+1} - \lambda _n| \le Ce^{-c|\lambda _n|^{\beta }}, \\&|\gamma _{n+1} - \gamma _n| \le Ce^{-c|\gamma _n|^{\beta '}}, \,\, \forall \, n \in \mathbb {Z}, \end{aligned}$$

where $\beta , \beta ' > 1$ and $C,c>0$ are two universal constants. The choice of $\pm c_i \log (1+n)^{\alpha }, \,\, \alpha \in (0,1),$ in the statement of Theorem 2 is mostly for aesthetic reasons.

The strategy in the proof of Theorem 2, given in Section 5.2, resembles some of the strategy in [22]. First, one obtains an *exponential decay* estimate for the solution at two different times through the fundamental theorem of calculus (see Lemma 4.7). We then use the result on propagation of exponential decay by Kenig, Ponce and Vega (see Lemma 5.1), in order to conclude that the same exponential decay estimate holds inside the time interval. We then use Lemma 4.4 in order to conclude that the solution is, in fact, *analytic* in a small strip of the complex plane. By a result on the zeros of analytic functions in a strip (Lemma 4.6), which is based on a theorem by Szegő, we impose constraints that are violated if $z_n = c_2 \log (1+n)^{\alpha }$ are our sequence of zeros. This yields a contradiction, which concludes the proof.

Our one dimensional result also generalizes to higher dimensions.

#### Theorem 3

Let $u \in C([0,T] :H^s(\mathbb {R}^d)),$ where $s > d/2 - 1$ be a strong solution to (2.5), where $d\ge 2$. Suppose additionally that $u_0, u(T) \in L^2( |x|^{2k} dx ),$ for some $k \in \mathbb {N}, k \ge \frac{d}{2},$ and that the solution satisfies

2.7 $$\begin{aligned} u_0|_{c_1 \log (1+n)^{\alpha } \cdot {\mathbb {S}}^{d-1}} = u(T)|_{c_2 \log (1+n)^{\alpha } \cdot {\mathbb {S}}^{d-1}} \equiv 0, \, \forall n \ge 0, \end{aligned}$$

in the sense of Sobolev traces for some $\alpha \in (0,1)$ and some $c_1,c_2>0$. Then $u \equiv 0.$

#### Remark 2.4

As before, by local smoothing of the Schrödinger equation (see Lemma 4.2), the assumptions $u_0,u(T) \in L^2(|x|^{2k} dx)$ guarantee that $u_0,u(T)\in H^1(\mathbb {R}^d)$ and are thus sufficient to make sense of the condition (2.7) in the sense of Sobolev traces.

The main strategy in the proof of Theorem 3, given in Section 6, is the same as that of the proof of Theorem 2. The main difference here is that the analogues to Lemma 4.7 and Lemma 4.4 are slightly more technical and somewhat weaker at times. At the end of the proof, however, we must use a slightly different approach: in order to conclude that the solution is identically zero, we restrict the initial (or final) datum to lines, and consider the restriction as an analytic function. By the structure of the zero set, we may find zeros that grow similarly to $c_2 \log (1+n)^{\alpha }$ for this new one-dimensional function, and thus the final part of the argument follows as well.

### Consequences for a Related Semilinear Elliptic Problem

As a consequence of the proof before, we notice the following. Fix $d \ge 2,$ and let $w \in H^{s}(\mathbb {R}^d) \cap L^2(\langle x \rangle ^{2k}), \, k \in \mathbb {N}, s > d/2 - 1, k \ge s$ be a solution to the following nonlinear scalar field equation:

2.8 $$\begin{aligned} \Delta w - w + \lambda w^3 = 0, \end{aligned}$$

where we fix $\lambda \in \mathbb {C}, \text {Re}(\lambda ) > 0.$ Let $u(x,t) = e^{it} w(x)$. It is immediate to see that *u* is a standing wave solution to the cubic NLS (2.5). Moreover, by the decay properties of *w*,  we see that $u \in C([0,T]; H^s(\mathbb {R}^d)), \, s > d/2 - 1$ satisfies the hypothesis of Theorem 3. Thus, we have the following Corollary as a direct consequence of that result:

#### Corollary 2

Let $w \in H^s(\mathbb {R}^d) \cap L^2(\langle x \rangle ^{2k}), s > \frac{d}{2}-1, k \in \mathbb {N}, k \ge d/2$ be a solution to (2.8), with $\text {Re}(\lambda ) > 0.$ Suppose that

$$\begin{aligned} w(c \log (n+1)^{\alpha } \xi )=0, \, \forall n \ge 0, \, \forall \xi \in {\mathbb {S}}^{d-1}. \end{aligned}$$

Then $$w \equiv 0.$$

As far as we know, this result is novel in the range $\alpha \in [3/4,1).$ For $\alpha \in (0,3/4),$ the result follows from the following result of Meshkov [18]: If *v* is a solution to $\Delta v + V \cdot v = 0$ in $\mathbb {R}^d$ with $v, V \in L^{\infty },$ and moreover $|v(x)| \lesssim e^{-c|x|^{4/3 + \varepsilon }},\,$ for some $c > 0$ and some $\varepsilon > 0,$ then $v \equiv 0.$

In our case, we take $V = \lambda w^2 - 1$ and $v = w.$ As *w* satisfies (2.8), it also satisfies, as noted above, (2.5). Referring to Lemma 4.2 in Section 4, we have $w \in H^{k,k}(\mathbb {R}^d), \, k \in \mathbb {N}, k \ge \frac{d}{2}$ (see already Section 3.2 for the definition of the space $H^{k,k}(\mathbb {R}^d)$). Now, already referring to the argument with Poincaré’s inequality in Lemma 4.9, we obtain that $|v(x)| \lesssim e^{-c|x|^{4/3+\varepsilon }},$ as long as $\alpha \in (0,3/4).$ One concludes then that *v* and *V* satisfy the hypotheses of Meshkov’s result above, and thus $v \equiv 0.$

One may, on the other hand, wonder whether Corollary 2 is sharp in any sense. We believe that this is not the case, and the reason is twofold.

First, the proof above was a mere application of Theorem 3 for the nonlinear Schrödinger equation, without taking too much into account the additional rigidity that equation (2.8) possesses. We believe that a method similar to that of [22], coupled with the Poincaré inequality trick from before, should lead to an improved version of that result. As this would already escape the scope of this manuscript, we plan to revisit this question in a future work.

Second, if one additionally imposes the condition that the solutions to (2.8) are *radial*, then much more can be said about the structure of zeros. Indeed, let $\lambda > 0$ in what follows. If $w = w(|x|)$ is twice differentiable (as a radial function) on the positive real line, equation (2.8) becomes

2.9 $$\begin{aligned} w'' + \frac{d-1}{r} w' + (\lambda w^2 - 1)\cdot w = 0, \, \forall \, r > 0. \end{aligned}$$

Equivalently, this may be rewritten as

2.10 $$\begin{aligned} (r^{d-1} w')' + r^{d-1}(\lambda w^2 - 1)\cdot w = 0, \, \forall \, r > 0. \end{aligned}$$

Suppose that $w \in L^{\infty }(\mathbb {R}^d).$ Let then $|\lambda \Vert w\Vert _{\infty }^2 - 1|= C.$ Fix ${\tilde{w}}$ a non-zero solution to the equation

$$\begin{aligned} (r^{d-1} {\tilde{w}}')' + C \cdot r^{d-1} {\tilde{w}} = 0, \, \forall \, r > 0. \end{aligned}$$

If we let $u(r) = r^{\frac{d-1}{2}} {\tilde{w}}\left( \frac{1}{\sqrt{C}} r\right) ,$ we see that *u* is a solution to Bessel’s equation

$$\begin{aligned} u'' + \left( 1 - \frac{\nu }{r^2} \right) u = 0, \end{aligned}$$

where $\nu = \frac{(d-1)(d-3)}{4}.$ Let $\{r_k\}_{k \in \mathbb {N}}$ be the (increasing) sequence of zeros of *w*. By the Sturm–Picone comparison principle, as *w* is not a constant, there is a sequence $\{r_k^1\}_{k \ge 1}$ of zeros of ${\tilde{w}}$ so that $r_k< r_k^1 < r_{k+1}, \, \forall \, k \ge 1.$ By considering the sequence $\{\sqrt{C}r_k^1\}$, we see that this is a sequence of zeros of the Bessel function *u* as above.

Fix an open interval *I* of length $\pi$ on the real line, with the center of *I* being sufficiently large in absolute value. We claim that there is at most one zero of *u* in *I*. Indeed, suppose not. That is, suppose we have two zeros $z_1, z_2$ of *u* in that interval. We then apply Sturm–Picone comparison once more: as $r \in I$ is sufficiently large in absolute value, we see that $1 - \frac{\nu }{r^2} < 1,$ and thus any solution of the equation

$$\begin{aligned} v'' + v = 0 \end{aligned}$$

has a zero in $(z_1,z_2) \subset I.$ If $I = (a,a+\pi ),$ then the function $v(t) = \sin (t-a)$ has no zeros in *I*,  a contradiction which proves the claim.

Therefore, the difference between (sufficiently large) consecutive zeros of *u* is at least $\ge \pi ,$ and thus concentrates at most like $\pi \mathbb {Z}$ near infinity. This shows that $c\log (1+n)^{\alpha }$ in Corollary 2 may be replaced by $(\pi -\varepsilon ) n,$ at least in the radial case.

Notice that, for the argument above, we did not need decay of *w*. Indeed, by using decay of the form $w \rightarrow 0$ in the argument above, one is able to obtain that any radial solution to (2.8) that decays at infinity does not change sign for $r > 0$ sufficiently large. We do not know whether this kind of behaviour can be extended to the non-radial setting. We do not investigate this question further here, however, as it would escape the scope of the manuscript, but we remark that this would be an interesting further question to pursue.

### Generalizations of the Main Results

In spite of the fact that this paper focuses on the question of uniqueness from zeros of the equation (2.5), one may generalize Theorem 2 and Theorem 3 to equations of the form

2.11 $$\begin{aligned} {\left\{ \begin{array}{ll} i \partial _t u = - \Delta u - \lambda |u|^{2k} u &{} \text { in } [0,T] \times \mathbb {R}^d, \\ u(x,0) = u_0(x) &{} \text { on } \mathbb {R}^d, \end{array}\right. } \end{aligned}$$

where $k \in \mathbb {N}$ is a positive integer. *Mutatis mutandis*, all propagation of regularity and analyticity results adapt with minor modifications in the exponents and techniques to this context. The final part of the proof itself can be adapted almost verbatim. In particular, the fact that we have restricted ourselves to the cubic case was due to the simpler technical aspect.

It is interesting to compare Theorem 1, Theorem 2 and Theorem 3 with the results in [7]. In the latter manuscript, the authors obtained uniqueness for the free Schrödinger equation (1.1) with zero sets behaving like $n^{\alpha },$ for some $\alpha > 0$ in a specific range. The zero sets in the assumptions of Theorem 1, Theorem 2 and Theorem 3 are significantly denser than that, and the reason for that is in the non-linear/potential effect.

First of all, while the free equation (1.1) may be explicitly given as a Fourier transform of the initial datum times a complex Gaussian, such a representation is not available in the non-linear case. Moreover, the same estimates which allow for the bootstrap argument in [22], which are implicitly used in [7], are seen to *fail* to improve decay in the non-linear case of Theorem 2 and Theorem 3. This has then to be compensated through a denser zero set, in order to enforce decay from the beginning. Similarly, in the case of Theorem 1, the decay needed for the bootstrap argument of [22] could be achieved by further assumptions on the potential. This, on the other hand, would immediately impose much more restrictive conditions on Corollary 1, a reason for which we decided to leave the question of obtaining a *sparser* zero set in Theorem 1 under additional decay on the potential to a future work.

This paper is structured as follows: in Section 3, we lay out the background knowledge and preliminaries. In Section 4 we prove certain smoothing and self-improving effects for the cubic Schrödinger equation based on our assumptions of the initial data and the solution at later times. Then, we show the main one-dimensional results in Section 5, and the higher-dimensional Theorem 3 in Section 6.

## Preliminaries and Basic Estimates

### Conventions

For the Fourier transform we use the convention ${\hat{f}}[\xi ] = {\mathcal {F}}(f) (\xi ) = \int _{\mathbb {R}^d} f(x) e^{-2\pi i \xi \cdot x} d x$. We will also denote generic constants with $C>0$ which may become larger from line to line and which may depend on the dimension *d*, on any Hölder, Strichartz, Besov exponents, on $\lambda$ as in (2.5), and on the fixed constants $c_1,c_2>0$ from the statements of the theorems. Similarly, we use $a\lesssim b$ if $a\le C b$ for a generic constant *C*.

### Function Spaces

We denote the standard Lebesgue spaces with $L^p(\mathbb {R}^d)$, the weighted Lebesgue spaces for a weight *w*(*x*) with $L^p( w(x) dx ) = L^p(\mathbb {R}^d, w(x) dx )$, and the Sobolev spaces with $W^{k,p}(\mathbb {R}^d)$ and the special case $H^k(\mathbb {R}^d) = W^{k,2}(\mathbb {R}^d)$. For $k \in {\mathbb {Z}}_{\ge 0}$ we define $H^{k,k}(\mathbb {R}^d)$ as the completion of the space of Schwartz functions under the norm $\Vert u \Vert _{H^{k,k}}^2 := \sum _{|\alpha | + |\beta | \le k} \Vert x^\alpha \partial _x^\beta u \Vert _{L^2(\mathbb {R}^d)}^2$ for multiindices $\alpha ,\beta \in {\mathbb {Z}}^d_{\ge 0}$. We will also say $\alpha \le \beta$ if $\alpha _i \le \beta _i$ for all $i = 1,\dots , d$ and we will use the convention that $\alpha < \beta$ means that $\alpha \le \beta$ and $\alpha \ne \beta$. We shall also use the notations $\Vert \cdot \Vert _p = \Vert \cdot \Vert _{L^p} = \Vert \cdot \Vert _{L^p(\mathbb {R}^d)}$ interchangeably. Similarly, for mixed spatial and temporal norms we also use the following notations $\Vert \cdot \Vert _{L^q([0,T]:L^r(\mathbb {R}^d))} = \Vert \cdot \Vert _{L^q_T L^r_x}=\Vert \cdot \Vert _{L^q L^r}$.

We will also make use of Besov spaces. To do so, we fix $\eta \in C_c^\infty (\mathbb {R}^d)$ with $\eta (\xi ) =1$ for $|\xi |\le 1$ and $\eta (\xi )=0$ for $|\xi |\ge 2$. For $j\in {\mathbb {Z}}$ we also define $\psi _j(\xi ) := \eta (\xi 2^{-j}) - \eta (\xi 2^{-j+1})$. Now, we define the Besov norm

3.1 $$\begin{aligned} \Vert u\Vert _{B^s_{p,q}(\mathbb {R}^d)} := \Vert {\mathcal {F}}^{-1} ( \eta {\hat{u}}) \Vert _{L^p(\mathbb {R}^d)} + {\left\{ \begin{array}{ll}\left( \sum _{j=1}^\infty ( 2^{sj} \Vert {\mathcal {F}}^{-1} (\psi _j {\hat{u}}) \Vert _{L^p(\mathbb {R}^d)} )^q\right) ^\frac{1}{q} &{} \text { if } q<\infty \\ \sup _{j\ge 1} 2^{sj} \Vert \mathcal {F}^{-1} (\psi _j {\hat{u}}) \Vert _{L^p(\mathbb {R}^d)} &{} \text { if } q=\infty \end{array}\right. } \end{aligned}$$

and denote with $B_{p,q}^s(\mathbb {R}^d)$ the space of tempered distribution $u\in {\mathcal {S}}'(\mathbb {R}^d)$ with $\Vert u\Vert _{B^s_{p,q}(\mathbb {R}^d)}<\infty$. We will mainly follow the convention that $p,q,r \in [1,\infty ]$, $s \in \mathbb {R}_{\ge 0}$, and $k\in {\mathbb {Z}}_{\ge 0}$.

### Strichartz Pairs and Estimates

We recall that a pair (*q*, *r*) of exponents is called admissible if $2\le q, r \le \infty$, $\frac{2}{q} + \frac{d}{r} = \frac{d}{2}$ and $(q,r,d) \ne (2,\infty ,2)$. Then, for any such pairs (*q*, *r*) and $({{\tilde{q}}}, {{\tilde{r}}})$, the following Strichartz estimates (e.g. [25, Theorem 2.3]) hold

3.2 $$\begin{aligned}&\Vert e^{it\Delta } u_0\Vert _{L^q L^r} \lesssim \Vert u_0\Vert _{L^2} \end{aligned}$$

3.3 $$\begin{aligned}&\Vert \int _{0}^t e^{i (t-\tau ) \Delta } F(\tau ) d \tau \Vert _{L^q L^r} \lesssim \Vert F\Vert _{L^{{{\tilde{q}}} '} L^{{{\tilde{r}}} '}}, \end{aligned}$$

where $( {{\tilde{q}}} ',{{\tilde{r}}}')$ are the Hölder conjugates of $({{\tilde{q}}}, {{\tilde{r}}})$.

### Notation and Basic Estimates

#### Lemma 3.1

For $f\in L^1( \langle x \rangle dx )$ denote by $v(x,t) = e^{it\partial _x^2}f(x)$ the solution of the *free Schrödinger equation*

3.4 $$\begin{aligned} {\left\{ \begin{array}{ll} i \partial _t v = -\partial _x^2 v, &{} \text { in } \mathbb {R}\times \mathbb {R}, \\ v(x,0) = f(x) &{} \text { in } \mathbb {R}. \\ \end{array}\right. } \end{aligned}$$

Then, for any $t>0$, the following estimate holds:

3.5 $$\begin{aligned} |e^{it\partial _x^2}f(x) - e^{it\partial _x^2}f(y)| \le C \min \left\{ \frac{ \max \{|x|,|y|\} |x-y| }{t^{3/2}} \Vert (1+|z|) f(z) \Vert _1, \frac{1}{t^{1/2}} \Vert f\Vert _1\right\} .\nonumber \\ \end{aligned}$$

#### Proof

Let $x,y \in \mathbb {R}$ be two arbitrary points. We remark the following formula for the solution of the free Schrödinger equation in one dimension:

$$\begin{aligned} e^{it\partial _x^2}f(z) = \frac{1}{(4 \pi i t)^{1/2}} \int _{\mathbb {R}} e^{\frac{i(z-y)^2}{4t}} f(y) \, dy. \end{aligned}$$

Then we have the following comparison estimate:

$$\begin{aligned} |e^{it\partial _x^2}f(x) - e^{it\partial _x^2}f(y)|&\le \frac{1}{(4\pi t)^{1/2}} \int _{\mathbb {R}} |e^{\frac{i(x-z)^2}{4t}} - e^{\frac{i(y-z)^2}{4t}}| \, |f(z)| \, dz \\&\le \frac{C}{t^{3/2}} \left( |x^2 - y^2| \Vert f\Vert _1 + |x-y| \Vert z f(z)\Vert _1 \right) \\&\le \frac{C \max \{|x|,|y|\} |x-y| }{t^{3/2}} \Vert (1+|z|) f(z) \Vert _1, \end{aligned}$$

for some absolute constant $C>0.$ On the other hand, by just using the triangle inequality, we have

$$\begin{aligned} \Vert e^{it\partial _x^2}f\Vert _{\infty } \le \frac{C}{t^{1/2}} \Vert f\Vert _1, \end{aligned}$$

for some possibly different absolute constant $C>0.$ This finishes the proof of the lemma. $\square$

### The Operator $\Gamma$

We now move on to analyse how regularity and decay relate to each other in the context of the cubic NLS. Indeed, we recall from [10] the operators

3.6 $$\begin{aligned} \Gamma ^\beta u(t) = \Gamma _t^\beta u(t) := e^{i|x|^2/4t} (2it)^{|\beta |} \partial _x^\beta (e^{-i|x|^2/4t} u(t)) = (x + 2it\partial _x)^\beta u(t) \end{aligned}$$

for a multiindex $\beta \in {\mathbb {Z}}_{\ge 0}^d$. It is straightforward to check that the operator $\Gamma _t^\beta$ commutes with the free Schrödinger operator:

3.7 $$\begin{aligned}{}[\Gamma _t^\beta , -i \partial _t - \Delta ] =0, \end{aligned}$$

and satisfies

3.8 $$\begin{aligned} \Gamma _t^\beta f = e^{it\Delta } x^\beta e^{-it\Delta } f \text { and } \Gamma _t^\beta e^{i s \Delta } f = e^{is\Delta } \Gamma ^\beta _{t-s} f. \end{aligned}$$

Moreover, for $|\alpha |=|\beta |=1$ we have the commutator relation

3.9 $$\begin{aligned}{}[\Gamma _{t}^\alpha , x^\beta ]= 2it \delta _{\alpha \beta }, \end{aligned}$$

where $\delta _{\alpha \beta }=1$ if $\alpha =\beta$ and $\delta _{\alpha \beta }=0$ if $\alpha \ne \beta$. In particular, for general multiindices $\alpha , \beta \in {\mathbb {Z}}_{\ge 0}^d$, a direct induction argument shows

3.10 $$\begin{aligned}{}[x^\beta , \Gamma _{t}^\alpha ]= \sum _{\begin{array}{c} {\tilde{\alpha }}< \alpha \\ {\tilde{\beta }}< \beta \end{array} } C_1 ({\tilde{\alpha }}, {\tilde{\beta }}, \alpha ,\beta , t) x^{{\tilde{\beta }}} \Gamma _t^{{\tilde{\alpha }}}, \;\; [x^{ \beta }, \Gamma _t^{ \alpha }] = \sum _{\begin{array}{c} {\tilde{\alpha }}< \alpha \\ {\tilde{\beta }} < \beta \end{array}} C_2 ({\tilde{\alpha }}, \tilde{\beta }, \alpha ,\beta , t) \Gamma _t^{{\tilde{\alpha }}}x^{{\tilde{\beta }}} \end{aligned}$$

for suitable $C_1$ and $C_2$.

### The Cubic NLS and Well-posedness

We now recall standard local well-posedness of (2.5) in the subcritical regime. We begin with the case of $d=1$ for $L^2(\mathbb {R})$ initial data.

#### Proposition 3.2

Let $u_0 \in L^2 ({\mathbb {R}})$. Then there exist a $T^*( \Vert u_0 \Vert _{L^2({\mathbb {R}}) } ) >0$ and a unique strong solution $u \in C([0,T^*);L^2({\mathbb {R}})) \cap L^4_{loc} ([0,T^*);L^\infty ({\mathbb {R}}))$ to (2.5). Moreover, $u\in L^q( [0,T]; L^r({\mathbb {R}}) )$ for any Strichartz pair (*q*, *r*) and every $T< T^*$.

In the case $d\ge 2$, the critical regularity obtained from scaling is

3.11 $$\begin{aligned} s_c =\frac{d}{2} -1\end{aligned}$$

and we have the following subcritical well-posedness.

#### Proposition 3.3

Let $d\ge 2$ and $u_0 \in H^s({\mathbb {R}}^d)$ for $s=s_c + \epsilon$ and some $\epsilon \in (0,1)$. Then there exist a $T^*( \Vert u_0\Vert _{H^s}) >0$ and a unique strong solution $u \in C([0,T^*);H^s({\mathbb {R}}^d)) \cap L_{loc}^\gamma ([0,T^*);B_{\rho ,2}^s ({\mathbb {R}}^d))$ to (2.5), where $\gamma :=\frac{8}{d-2s}$ and $\rho :=\frac{4d}{d+2s}$. Moreover,

$$\begin{aligned} u\in L^q( [0,T]; B_{r,2}^{s}({\mathbb {R}}^d) ) \end{aligned}$$

for any Strichartz pair (*q*, *r*) and every $T< T^*$.

We also remark (see e.g. [2, Section 1.6]) that strong solutions to (2.5) are equivalently characterized by the associated Duhamel formulation of cubic nonlinear Schrödinger equation

3.12 $$\begin{aligned} u(t) = e^{it\Delta } u_0 +i \lambda \int _0^t e^{i \Delta (t-\tau )} |u|^2 u(\tau ) d \tau . \end{aligned}$$

## Self-improving of Decay and Regularity for the Cubic NLS

We begin this section with two lemmas that guarantee that solutions to the cubic Schrödinger equation are smooth and have decay measured by weighted Sobolev spaces, if decay is only known at two different times.

### Decay and Regularity in Weighted Sobolev Spaces

The results in this subsection may be considered folklore, see [8–10] and [2, Chapter 5.6] for similar results. We have however decided to lay out the details in order to keep the exposition self-contained, aiming at being a unified reference for those results. We hope that, by doing so, this also improves the readability of the paper. We begin with the result for the case $d=1$.

#### Lemma 4.1

Let $u_0 \in L^2({\mathbb {R}})$ and let $u \in C([0,T] :L^2(\mathbb {R})) \cap L^4([0,T] :L^{\infty }(\mathbb {R}))$ for $T< T^*(\Vert u_0\Vert _{L^2({\mathbb {R}})} )$ be the unique strong solution to the cubic Schrödinger equation (2.5) as defined in Proposition 3.2.

If in addition $u_0, u(T) \in L^2(x^{2k} \, dx)$ for some $k\in {\mathbb {N}}$, then $u \in C([0,T] :H^k(\mathbb {R}) \cap L^2(\langle x \rangle ^{2k} \, dx) ).$

In higher dimensions, we have the following result, where we recall that $s_c = \frac{d}{2} -1$ from (3.11).

#### Lemma 4.2

Let $d \ge 2$ and $u_0 \in H^s({\mathbb {R}}^d)$ for some $s=s_c + \epsilon$ and $\epsilon \in (0,1)$. Let $u \in C([0,T];H^s({\mathbb {R}}^d)) \cap L^\gamma ([0,T];B_{\rho ,2}^s ({\mathbb {R}}^d))$ for $T< T^*(\Vert u_0\Vert _{H^s({\mathbb {R}})} )$ be the unique strong solution to the cubic Schrödinger equation (2.5) as defined in Proposition 3.3.

If in addition $u_0, u(T) \in L^2(\langle x \rangle ^{2k} \, dx)$ for some $k\in {\mathbb {N}}$, $k\ge s$, then $u \in C([0,T] :H^k(\mathbb {R}^d) \cap L^2(\langle x \rangle ^{2k} \, dx) ).$

#### Proof of Lemmata 4.1 and 4.2

We shall break the proof into several smaller steps.

**Step 1: Proving** $\Gamma ^\alpha u \in C([0,T]:L^2({\mathbb {R}}^d))$ **for** $|\alpha | =k$. Let $u_{0,n}$ be a sequence of Schwartz functions with $u_{0,n} \rightarrow u_0$ in $L^2(\langle x \rangle ^{2k} dx ) \cap H^s({\mathbb {R}}^d)$ as $n\rightarrow \infty$. Let $u_n$ be the sequence of corresponding solutions to (3.12), i.e.

4.1 $$\begin{aligned} u_n(t,x) = e^{it\Delta }u_{0,n}(x) + i\lambda \int _{0}^{t} e^{i(t-\tau )\Delta } |u_n|^2 u_n(\tau ,x) d \tau . \end{aligned}$$

For $|\alpha |= k$ we apply $\Gamma _t^\alpha$ on both sides and obtain

4.2 $$\begin{aligned} \Gamma ^\alpha _t u_n = e^{it\Delta } x^\alpha u_{0,n} +i\lambda \int _0^t e^{i(t-\tau ) \Delta } \Gamma _\tau ^\alpha ( |u_n|^2 u_n(\tau ,x)) d \tau \end{aligned}$$

in view of $\Gamma _t^\alpha e^{it\Delta } = e^{it \Delta } x^\alpha$.

Taking the spatial $L^2$-norm of (4.2) together with the fact that $\Vert e^{it\Delta } f\Vert _{L^2} = \Vert f\Vert _{L^2}$ yields

4.3 $$\begin{aligned} \Vert \Gamma _t^\alpha u_n \Vert _{L^2} (t) \le \Vert x^\alpha u_{0,n}\Vert _{L^2} + |\lambda | \int _0^t \Vert \Gamma _\tau ^\alpha (|u_n|^2 u_n)\Vert _{L^2} (\tau )\mathrm {d}\tau . \end{aligned}$$

We will now estimate $\Gamma _\tau ^\alpha (|u_n|^2 u_n)$. To do so we set ${{\tilde{u}}}_n := e^{ - i\frac{x^2}{4\tau }} u_n$ such that

4.4 $$\begin{aligned} \Gamma _\tau ^\alpha (|u_n|^2 u_n) = \Gamma _\tau ^\alpha \left( e^{i\frac{x^2}{4\tau }} |{{\tilde{u}}}_n|^2 {{\tilde{u}}}_n \right) = e^{i\frac{x^2}{4\tau }} (2i \tau \partial _x)^\alpha \left( |\tilde{u}_n|^2 {{\tilde{u}}}_n \right) . \end{aligned}$$

Now, using the Gagliardo–Nirenberg interpolation inequality we obtain

4.5 $$\begin{aligned} \Vert \Gamma _\tau ^\alpha (|u_n|^2 u_n)\Vert _{L^2(\mathbb {R}^d) }&\lesssim \tau ^k \Vert \partial _x^\alpha (|{{\tilde{u}}}_n|^2 {{\tilde{u}}}_n)\Vert _{L^2(\mathbb {R}^d)} \nonumber \\&\lesssim \tau ^k \Vert \partial _x^\alpha {{\tilde{u}}}_n \Vert _{L^2(\mathbb {R}^d)} \Vert {{\tilde{u}}}\Vert _{L^\infty (\mathbb {R}^d)}^2 \lesssim \Vert \Gamma _\tau ^\alpha u_n \Vert _{L^2(\mathbb {R}^d)} \Vert u\Vert _{L^\infty (\mathbb {R}^d)}^2 . \end{aligned}$$

Using the above estimate and applying Gronwall’s lemma to (4.3) gives

4.6 $$\begin{aligned} \Vert \Gamma ^\alpha _t u_n\Vert _{L^2(\mathbb {R}^d)} (t)\le \Vert x^\alpha u_{0,n} \Vert _{L^2(\mathbb {R}^d)} \exp \left( |\lambda | \int _0^t \Vert u_n \Vert _{L^\infty (\mathbb {R}^d)}^2(\tau ) \ d\tau \right) . \end{aligned}$$

Since $u_{0,n}\rightarrow u_0$ in $L^2( \langle x \rangle ^{2k}dx ) \cap H^s(\mathbb {R}^d)$, we have $\Vert x^\alpha u_{0,n}\Vert \rightarrow \Vert x^\alpha u_0\Vert$ as $n \rightarrow \infty$. Thus, in order to obtain uniform control over $\Vert \Gamma ^k_t u_n\Vert _{L^2(\mathbb {R}^d)}$ we have to control

$$\begin{aligned} \int _0^t \Vert u_n \Vert _{L^\infty (\mathbb {R}^d)}^2(\tau ) \ d\tau . \end{aligned}$$

We first consider the case $d=1$. In this case we have from well-posedness in Proposition 3.2 that $u_n \rightarrow u$ in $C([0,T] :L^2(\mathbb {R})) \cap L^4([0,T] :L^{\infty }(\mathbb {R})),$ which shows that

$$\begin{aligned} \int _0^t \Vert u_n \Vert _{L^\infty (\mathbb {R})}^2(\tau ) \ d\tau \le t^{\frac{1}{2}} \Vert u_n\Vert _{L^4([0,T] :L^{\infty }(\mathbb {R}))}^2, \end{aligned}$$

and the term on the right-hand side is uniformly bounded.

The case $d\ge 2$ is slightly more involved. We first use the Gagliardo–Nirenberg interpolation inequality estimate

4.7 $$\begin{aligned} \Vert u_n \Vert _{L^\infty (\mathbb {R}^d) } \lesssim \Vert u_n \Vert _{W^{s,r}(\mathbb {R}^d)} \end{aligned}$$

for $r= \frac{d}{s} = \frac{2d}{d-2+ 2 \epsilon }$. The corresponding temporal Strichartz exponent is $q=\frac{2}{1-\epsilon }$. Hence,

4.8 $$\begin{aligned} \int _0^t \Vert u_n \Vert _{L^\infty (\mathbb {R}^d)}^2(\tau ) d\tau&= \Vert u_n\Vert _{L^2([0,T]: L^\infty (\mathbb {R}^d))}^2 \nonumber \\ \lesssim \Vert u_n\Vert _{L^2([0,T]: W^{s,r}(\mathbb {R}^d))}^2&\lesssim T^\epsilon \Vert u_n\Vert _{L^{\frac{2}{1-\epsilon }} ([0,T]: W^{s,r}(\mathbb {R}^d))}^2. \end{aligned}$$

Using the embedding $B^{s}_{r,2}(\mathbb {R}^d) \hookrightarrow W^{s,r}(\mathbb {R}^d)$, $r\ge 2$ (see e.g. [1, Theorem 6.4.5]) we then obtain from well-posedness in Proposition 3.3 the uniform control over $\int _0^t \Vert u_n \Vert _{L^\infty (\mathbb {R}^d)}^2(\tau ) d\tau$.

Thus, we have shown in both cases, $d=1$ and $d\ge 2$, that the following a priori bound holds

4.9 $$\begin{aligned} \sup _{n} \sup _{0\le t \le T} \Vert \Gamma _t^\alpha u_n \Vert _{L^2(\mathbb {R}^d)}(t) \le \Vert x^\alpha u_0\Vert _{L^2(\mathbb {R}^d)} \exp \left( |\lambda | \int _0^T \Vert u\Vert _{L^\infty (\mathbb {R}^d)}^2 d \tau \right) . \end{aligned}$$

We will now show that $(\Gamma _t^\alpha u_n)_n$ is a Cauchy sequence in $C([0,T]: L^2(\mathbb {R}^d))$. We begin by estimating

4.10 $$\begin{aligned} \Vert \Gamma _t^\alpha u_n - \Gamma _t^\alpha u_m \Vert _{L^2(\mathbb {R}^d)} \le&\Vert x^\alpha (u_{0,n} - u_{0,m}) \Vert _{L^2(\mathbb {R}^d)} \nonumber \\&+ \int _0^t \Vert \Gamma _ \tau ^\alpha ( u_n |u_n|^2 - u_m |u_m|^2 ) \Vert _{L^2(\mathbb {R}^d)}( \tau ) d \tau . \end{aligned}$$

In order to control the nonlinearity we use the following estimate which is similar to (4.5):

$$\begin{aligned} \Vert \Gamma _ \tau ^\alpha&( u_n |u_n|^2 - u_m |u_m|^2 ) \Vert _{L^2(\mathbb {R}^d)} \lesssim \Big [ \left( \Vert \tau ^k \partial _x^\alpha ({{\tilde{u}}}_n - {{\tilde{u}}}_m ) \Vert _{L^2 (\mathbb {R}^d)} + \Vert \tau ^k ({{\tilde{u}}}_n - {{\tilde{u}}}_m ) \Vert _{L^2(\mathbb {R}^d) } \right) \\&\cdot \left( \Vert \tilde{u}_n \Vert _{L^\infty (\mathbb {R}^d)}^2 + \Vert {{\tilde{u}}}_m \Vert _{L^\infty (\mathbb {R}^d)}^2 \right) \Big ] + \Big [ \Vert {{\tilde{u}}}_n - {{\tilde{u}}}_m\Vert _{L^\infty } \left( \Vert {{\tilde{u}}}_n \Vert _{L^\infty (\mathbb {R}^d)}+ \Vert {{\tilde{u}}}_m \Vert _{L^\infty (\mathbb {R}^d)} \right) \\&\cdot \left( \Vert \tau ^k \partial _x^\alpha {{\tilde{u}}}_n \Vert _{L^2(\mathbb {R}^d)} + \Vert \tau ^k {{\tilde{u}}}_n \Vert _{L^2(\mathbb {R}^d)} + \Vert \tau ^k \partial _x^\alpha {{\tilde{u}}}_m \Vert _{L^2(\mathbb {R}^d)} + \Vert \tau ^k {{\tilde{u}}}_m \Vert _{L^2(\mathbb {R}^d)} \right) \Big ]\\ \lesssim&\left( \Vert \Gamma ^\alpha _\tau (u_n -u_m)\Vert _{L^2(\mathbb {R}^d)} + \tau ^k \Vert u_n - u_m \Vert _{L^2(\mathbb {R}^d)} \right) \left( \Vert u_n \Vert _{L^\infty (\mathbb {R}^d)}^2 + \Vert u_m \Vert _{L^\infty (\mathbb {R}^d)}^2 \right) \\&+\Big [ \Vert u_n - u_m\Vert _{L^\infty (\mathbb {R}^d) } \left( \Vert u_n \Vert _{L^\infty (\mathbb {R}^d)} + \Vert u_m \Vert _{L^\infty (\mathbb {R}^d)} \right) \\&\cdot \left( \Vert \Gamma _\tau ^\alpha u_n \Vert _{L^2} + \Vert \tau ^k u_n \Vert _{L^2} + \Vert \Gamma _\tau ^\alpha u_m \Vert _{L^2(\mathbb {R}^d)} + \Vert \tau ^k u_m \Vert _{L^2(\mathbb {R}^d)} \right) \Big ].&\end{aligned}$$

Inserting the above in the right-hand side of (4.10) and using the uniform bounds on $\Vert u_n\Vert _{L^2([0,T]: L^\infty (\mathbb {R}^d)) }, \Vert u_n\Vert _{L^\infty ([0,T]: L^2(\mathbb {R}^d)) }$, $\Vert \Gamma ^\alpha _t u_n\Vert _{L^\infty ([0,T]: L^2(\mathbb {R}^d))}$ we obtain

4.11 $$\begin{aligned} \Vert \Gamma _t^\alpha ( u_n - u_m ) \Vert _{L^2(\mathbb {R}^d)}&\lesssim \Vert x^\alpha (u_{0,n} - u_{0,m}) \Vert _{L^2(\mathbb {R}^d)} \nonumber \\&+ {{\tilde{C}}} \int _0^t \Vert \Gamma _t^\alpha (u_n-u_m) \Vert _{L^2(\mathbb {R}^d)} \nonumber \\&+ \Vert u_n - u_m\Vert _{L^\infty (\mathbb {R}^d)}^2 + \Vert u_n - u_m\Vert _{L^2(\mathbb {R}^d)} d \tau , \end{aligned}$$

for some ${{\tilde{C}}}= {{\tilde{C}}}(\Vert u_0 \Vert _{L^2(\langle x \rangle ^{2k}dx)} , \Vert u_0\Vert _{H^s} )>0$. Using Gronwall’s lemma, together with the fact that

$$\begin{aligned} \sup _t \Vert x^\alpha (u_{0,n} - u_{0,m}) \Vert _{L^2 (\mathbb {R}^d)} \rightarrow 0,&\int _0^T \Vert u_n - u_m\Vert _{L^\infty (\mathbb {R}^d)}^2 d \tau \rightarrow 0, \\&\int _0^T \Vert u_n - u_m\Vert _{L^2(\mathbb {R}^d) } d \tau \rightarrow 0 \end{aligned}$$

as $n,m\rightarrow \infty$, shows that $\Gamma _t^\alpha u_n$ is a Cauchy sequence in $C([0,T];L^2(\mathbb {R}^d))$. As $u_n \rightarrow u$ in $C([0,T];L^2(\mathbb {R}^d))$, we conclude that $\Gamma _t^\alpha u$ is a well-defined $L^2({\mathbb {R}}^d)$ function for each $t\in [0,T]$ and indeed $\Gamma _t^\alpha u_n \rightarrow \Gamma _t^\alpha u$ in $C([0,T];L^2(\mathbb {R}^d))$ with the bound

4.12 $$\begin{aligned} \sup _{0\le \tau \le t} \Vert \Gamma _\tau ^\alpha u \Vert _{L^2(\mathbb {R}^d)} \le \Vert x^k u_0\Vert _{L^2(\mathbb {R}^d)} \exp \left( |\lambda | \int _0^t \Vert u\Vert _{L^\infty (\mathbb {R}^d)}^2 d \tau \right) . \end{aligned}$$

This concludes Step 1.

**Step 2: Showing that** $u_0, u(T) \in H^{k,k}({\mathbb {R}}^d)$. From Step 1 and the assumption of the lemma, we have $\Gamma ^\alpha u (T)\in L^2({\mathbb {R}}^d)$ and $x^\alpha u(T) \in L^2 ({\mathbb {R}}^d)$ for $|\alpha |\le k$. In particular, this shows that $\partial _x^\alpha {{\tilde{u}}}(T) \in L^2({\mathbb {R}}^d)$ and $x^\alpha {{\tilde{u}}}(T) \in L^2({\mathbb {R}}^d)$ for $|\alpha |\le k$, where we recall that ${{\tilde{u}}}(T) = e^{-i \frac{x^2}{4 T}} u(T)$.

In order to continue, we will need an auxiliary interpolation result, which appears as Lemma 4.3 below. With Lemma 4.3 we now conclude that $\tilde{u}(T) \in H^{k,k}$. In particular, this shows that $\sum _{|\alpha |+|\beta |\le k} \Vert x^\alpha \Gamma ^\beta _T u(T)\Vert _{L^2(\mathbb {R}^d)} <\infty$. To conclude that $u(T)\in H^{k,k}(\mathbb {R}^d),$ we expand the differentiation operator of order $\beta$ as

$$\begin{aligned} \partial _x^\alpha = \sum _{\beta +\gamma \le \alpha } C(\alpha ,\beta ,\gamma ,T) x^{\beta } \Gamma _T^\gamma . \end{aligned}$$

Thus,

4.13 $$\begin{aligned} \sum _{|\alpha | + |\beta | \le k} \Vert x^\alpha \partial _x^\beta u(T)\Vert _{L^2(\mathbb {R}^d)} < \infty \end{aligned}$$

and $u(T)\in H^{k,k}(\mathbb {R}^d)\subset H^k(\mathbb {R}^d)$. Completely analogously we have $u_0 \in H^{k,k}(\mathbb {R}^d)\subset H^{k}(\mathbb {R}^d)$ and by a standard persistence of regularity result (analogous to Step 1 above, see e.g. [2, Chapter 5]) in $H^k(\mathbb {R}^d)$, $k\ge 0$, we have $u\in C([0,T] :H^{k}(\mathbb {R}^d))$, where we used for $d=1$, $k\ge s$ for $d\ge 2$. Again, using the interpolation result from Lemma 4.3 for each $t\in [0,T]$, we finally obtain $u\in C([0,T] :H^{k}(\mathbb {R}^d)\cap L^2(\langle x \rangle ^{2k} dx) )$. This concludes the proof.

$$\square $$

As Lemma 4.3 was a crucial ingredient in the proof above, we will provide a proof for it below. The result can be originally found in a previous work of H. Triebel [26], where the author employs tools from the theory of interpolation spaces to prove such a theorem. In the present case, however, a much more direct proof is available, which we sketch below.

#### Lemma 4.3

Let $f \in L^2(\mathbb {R}^d)$ satisfy that $\Vert |x|^j f\Vert _{L^2(\mathbb {R}^d)} + \Vert \partial _x^{\alpha } f\Vert _{L^2(\mathbb {R}^d)} < +\infty$ for $j,|\alpha |\le k,$ for some positive integer $k \ge 1.$ Then we have that

$$\begin{aligned} \sum _{l+ |\beta | \le k} \Vert |x|^{l} \partial _x^{\beta } f\Vert _{ L^2(\mathbb {R}^d)} \lesssim \sum _{j,|\alpha |\le k} (\Vert |x|^j f \Vert _{L^2(\mathbb {R}^d)} + \Vert \partial _x^{\alpha } f\Vert _{L^2(\mathbb {R}^d)}). \end{aligned}$$

#### Proof

(Sketch of proof) We argue by induction on *k*. For $k =1 ,$ the result is tautological. For $k=2,$ we only need to prove that $\Vert x_i \partial _{x_j} f\Vert _2 < + \infty ,$ for $i,j \in \{1,\dots ,d\}.$ But

$$\begin{aligned} \Vert x_i \partial _{x_j} f\Vert _2^2&= \int _{\mathbb {R}^d} x_i^2 \partial _{x_j} f \overline{\partial _{x_j} f} \, dx \\&= - \int _{\mathbb {R}^d} x_i^2 \partial _{x_j}^2 f \cdot \overline{f(x)} \, dx - 2 \delta _{i,j} \int _{\mathbb {R}^d} x_i \partial _{x_j}f \overline{f(x)}\, dx \\&\lesssim \Vert \partial _{x_j}^2 f\Vert _2 \Vert |x|^2 f\Vert _2 + \Vert |x| f\Vert _2\Vert \partial _{x_j}f\Vert _2. \end{aligned}$$

This shows the result in that case.

Now, for $k \ge 3,$ we suppose the result holds for $j = 1,\dots ,k-1$ and fix $\beta$ a multiindex of integers, $l \in \mathbb {N}$ so that $l + |\beta | = k$. Select $i \in \{1,\dots ,d\}$ so that $\beta _i > 0.$ Let also $e_i$ denote the multiindex whose $j-$th coordinate is $\delta _{i,j}.$ Then we may write

$$\begin{aligned} \Vert |x|^{l} \partial _x^{\beta } f\Vert _2 = \left\| |x|^l \partial _x^{\beta -e_i} (\partial _x^{e_i} f) \right\| _2. \end{aligned}$$

By induction hypothesis applied to the function $g := \partial _x^{e_i} f$, we have that the latter term on the right-hand side above is bounded by

$$\begin{aligned} \sum _{j,|\alpha | \le k-1} \left( \Vert |x|^{j} \partial _x^{e_i} f\Vert _2 + \Vert \partial _x^{\alpha } (\partial _x^{e_i} f)\Vert _2 \right) . \end{aligned}$$

Notice that the second terms in the summand are all included in $\sum _{|\alpha | \le k} \Vert \partial _x^{\alpha } f\Vert _2,$ and so we focus on the first term. Indeed, if $j \le k-2,$ we may use the induction hypothesis again, and we are thus only concerned with estimating $\Vert |x|^{k-1} \partial _{x_i} f \Vert _2.$ Analogously as in the $k=2$ case, we have

$$\begin{aligned} \Vert |x|^{k-1} \partial _{x_i} f \Vert _2^2&\lesssim \int _{\mathbb {R}^d} |x|^{2k-3} |\partial _{x_i} f| |f| + \int _{\mathbb {R}^d} |x|^{2k-2} |\partial _{x_i}^2 f| |f| \\&\lesssim \Vert |x|^{k-2} \partial _{x_i} f\Vert _2 \Vert |x|^{k-1} f\Vert _2 + \Vert |x|^{k-2} \partial _{x_i}^2 f \Vert _2 \Vert |x|^k f \Vert _2. \end{aligned}$$

We now use the same trick once more: we may write

$$\begin{aligned} \Vert |x|^{k-2} \partial _{x_i}^2 f \Vert _2 = \Vert |x|^{k-2} \partial _{x_i} (\partial _{x_i} f) \Vert _2, \end{aligned}$$

and again, by the induction hypothesis applied to $g:= \partial _{x_j} f,$ we get that the term above is bounded by

$$\begin{aligned} \sum _{l, |\alpha | \le k-1} \left( \Vert |x|^{l} \partial _{x_i} f \Vert _2 + \Vert \partial ^{\alpha + e_i} f\Vert _2 \right) . \end{aligned}$$

Notice that the only term in the bound above that is not controlled by either induction or by the quantity in the statement is *exactly* $\Vert |x|^{k-1} \partial _{x_i} f\Vert _2.$ For shortness, let

$$\begin{aligned} C_k(f) := \sum _{l,|\alpha | \le k} \left( \Vert |x|^l f\Vert _2 + \Vert \partial ^{\alpha } f\Vert _2 \right) . \end{aligned}$$

Putting together all we did so far, we obtain

$$\begin{aligned} \Vert |x|^{k-1} \partial _{x_i} f \Vert _2^2&\lesssim C_k^2 + C_k \left( \Vert |x|^{k-1} \partial _{x_i} f\Vert _2 + C_k\right) . \end{aligned}$$

This clearly implies that

$$\begin{aligned} \Vert |x|^{k-1} \partial _{x_i} f \Vert _2 \lesssim C_k, \end{aligned}$$

and thus the induction closes, and we are done. $\square$

### Analyticity and the Flow of the Cubic NLS

In this subsection, we will prove some properties on analyticity and local well-posedness in some spaces of analytic function, as first considered by Hayashi and Saitoh [11, 12]. In particular, we will follow closely the strategy used in [13] by Hoshino and Ozawa, where the authors analyse the case of the quintic NLS in one dimension.

We state some definitions in order to prove the next Lemma. Indeed, first we define the class $A_2(r)$ as

4.14 $$\begin{aligned} A_2(r) = \left\{ \phi \in L^2(\mathbb {R}) :\Vert \phi \Vert _{A_2(r)} = \sum _{j \ge 0} \frac{r^j}{j!} \Vert x^j \phi \Vert _2 < + \infty \right\} . \end{aligned}$$

By [20, Proposition 2] (see also [19, Remark 3]), this norm can be bounded as

$$\begin{aligned} \Vert e^{r|x|} \phi (x) \Vert _2 \le \Vert \phi \Vert _{A_2(r)} \le (1+2 \log 2)^{\frac{1}{2}} \Vert (1+r|x|)^{1/2} e^{r|x|} \phi (x) \Vert _2. \end{aligned}$$

Moreover, we define the spaces

4.15 $$\begin{aligned} {\mathcal {A}}_{p,q}(r;T) = \left\{ u \in L^{\infty }_T L^2_x :\Vert u\Vert _{{\mathcal {A}}(r;T)} = \sum _{j \ge 0} \frac{r^j}{j!} \Vert \Gamma ^j u\Vert _{L^{p}_T L^q_x} < +\infty \right\} . \end{aligned}$$

We state our next result in terms of persistence in this last space, which, as we shall see afterwards, may be translated into a result on analyticity of solutions of the cubic nonlinear Schrödinger equation (2.5).

#### Lemma 4.4

Let $u_0 \in A_2(r),$ for some $r>0.$ Then there is $T = T(\Vert u_0\Vert _{A_2(r)})$ such that the initial value problem (2.5) has a unique solution

$$\begin{aligned} u \in {\mathcal {A}}_{\infty ,2}(r;T) \cap {\mathcal {A}}_{8,4}(r;T) =: X(r;T). \end{aligned}$$

Moreover, the flow map $u_0 \mapsto u(t)$ is locally Lipschitz continuous from $A_2(r)$ to ${\mathcal {A}}_{\infty ,2}(r;T).$

#### Proof

We will use a Banach fixed-point argument. Indeed, we define the map

$$\begin{aligned} \Phi (u) = e^{it\Delta }u_0+ i\lambda \int _0^t e^{i(t-\tau )\Delta } (|u|^2 u)(\tau ) \, d\tau , \end{aligned}$$

and we wish to prove that this map is a contraction in some set

$$\begin{aligned} B(u_0) = \{ u \in X(r;T) :\Vert u\Vert _{X(r;T)} \le 2C\Vert u_0\Vert _{A_2(r)}\}. \end{aligned}$$

In order to do so, we notice that

4.16 $$\begin{aligned} \Gamma ^{j}(\Phi (u)) = e^{it\Delta }(x^j u_0) + i \lambda \int _0^t e^{i(t-\tau )\Delta } (\Gamma _\tau ^j (|u|^2 u)(\tau )) \, d\tau , \end{aligned}$$

for $j\in {\mathbb {N}}$ and moreover, using the properties of $\Gamma$ from (3.6),

4.17 $$\begin{aligned} \Gamma ^j(|u|^2u) = \sum _{k_1 + k_2 + k_3 = j} \frac{j!(-1)^{k_3}}{(k_1)! (k_2)! (k_3)!} \Gamma ^{k_1} u \cdot \Gamma ^{k_2} u \cdot \overline{ \Gamma ^{k_3} u}. \end{aligned}$$

By using the usual Strichartz estimates from (3.2) and (3.3), we obtain that

$$\begin{aligned} \Vert \Gamma ^j(\Phi (u))\Vert _{L^{\infty }_T L^2_x}&+ \Vert \Gamma ^j(\Phi (u))\Vert _{L^8_T L^4_x} \lesssim \Vert x^j u_0\Vert _2 \\&+ \sum _{k_1 + k_2 + k_3 = j} \frac{j!}{(k_1)! (k_2)! (k_3)!} \Vert \Gamma ^{k_1} u \cdot \Gamma ^{k_2} u \cdot \overline{ \Gamma ^{k_3} u}\Vert _{L^{p'}_T L^{q'}_x}, \end{aligned}$$

where (*p*, *q*) are a Strichartz pair of exponents and $p', q'$ are their respective Hölder conjugates. We choose $p=8,q=4.$ By Hölder’s inequality, we have

$$\begin{aligned} \Vert \Gamma ^j(\Phi (u))\Vert _{L^{\infty }_T L^2_x}&+ \Vert \Gamma ^j(\Phi (u))\Vert _{L^8_T L^4_x} \lesssim \Vert x^j u_0\Vert _2 \\&+\sum _{k_1 + k_2 + k_3 = j} \frac{j!}{(k_1)! (k_2)! (k_3)!} \Vert \Gamma ^{k_1} u\Vert _{L^{24/7}_T L^4_x} \cdot \Vert \Gamma ^{k_2} u\Vert _{L^{24/7}_T L^4_x} \cdot \Vert \Gamma ^{k_3} u\Vert _{L^{24/7}_T L^{4}_x}. \\&\lesssim \Vert x^j u_0\Vert _2 \\&+ T^{\omega } \sum _{k_1 + k_2 + k_3 = j} \frac{j!}{(k_1)! (k_2)! (k_3)!} \Vert \Gamma ^{k_1} u\Vert _{L^8_T L^4_x} \cdot \Vert \Gamma ^{k_2} u\Vert _{L^8_T L^4_x} \cdot \Vert \Gamma ^{k_3} u\Vert _{L^8_T L^{4}_x}, \end{aligned}$$

for some positive constant $\omega >0.$ By multiplying this estimate by $\frac{r^j}{j!}$ and summing up on $j \ge 0,$ we obtain

$$\begin{aligned} \Vert \Phi (u)\Vert _{X(r;T)} \le C\Vert u_0\Vert _{A_2(r)}+ CT^{\omega }\Vert u\Vert _{X(r;T)}^3. \end{aligned}$$

In the same way, for $u,v \in B(u_0)$ we obtain

$$\begin{aligned} \Vert \Phi (u) - \Phi (v)\Vert _{X(r;T)} \le C T^{\omega } (\Vert v\Vert _{X(r;T)}^2 + \Vert u\Vert _{X(r;T)}^2) \Vert u-v\Vert _{X(r;T)}. \end{aligned}$$

Thus, for $T \le \frac{1}{C'\Vert u_0\Vert _{A_2(r)}^2},$ for $C'$ sufficiently large, $\Phi$ becomes a contraction in $B(u_0),$ and thus we are able to conclude the claim. The claim about Lipschitz continuity follows in a similar manner. $\square$

In analogy to the lemmata 4.1 and 4.2, we present a higher-dimensional version of the last lemma, whose formulation is slightly weaker. In order to state it, we define a generalization of the spaces $A_2(r)$ above, where regularity is included. Indeed, we define the class $A_2^k(r)$ as

$$\begin{aligned} A_2^k(r) = \left\{ \phi \in L^2(\mathbb {R}^d) :\Vert \phi \Vert _{A_s^k(r)} = \sum _{j \in (\mathbb {Z}_{\ge 0})^d} \frac{r^{|j|}}{j!} \Vert x^j \phi \Vert _{2,k} < + \infty \right\} , \end{aligned}$$

where we use the notation $\Vert f \Vert _{2,k} = \sum _{|\alpha | \le k} \Vert \partial _x^{\alpha } f\Vert _2$ for a modification of the $H^k$ norm. Once more in analogy to the one dimensional case, we define the time-dependent space where we will prove that our solution lies as follows.

$$\begin{aligned} {\mathcal {A}}_{\infty ,2}^k(r,T) = \left\{ u \in L^{\infty }_T L^2_x :\Vert u\Vert _{{\mathcal {A}}^k(r;T)} = \sum _{j \in (\mathbb {Z}_{\ge 0})^d} \frac{r^{|j|}}{j!} \Vert \Vert \Gamma ^j u\Vert _{2,k}\Vert _{L^{\infty }_T} < +\infty \right\} . \end{aligned}$$

#### Lemma 4.5

Let $k > \frac{d}{2}$ be a positive integer, and $u_0 \in A_2^k(r).$ Then there is $T = T(\Vert u_0\Vert _{A_2^k(r)})$ such that the initial value problem (2.5) has a unique solution

$$\begin{aligned} u \in {\mathcal {A}}_{\infty ,2}^k(r,T). \end{aligned}$$

Moreover, the flow map $u_0 \mapsto u(t)$ is locally Lipschitz continuous from $A_2^k(r)$ to ${\mathcal {A}}_{\infty ,2}^k(r,T).$

#### Proof

We will use, once more, a Banach fixed-point argument. Indeed, consider again the map $\Phi (u)$ from the proof of Lemma 4.4. We wish to prove that this map is a contraction in some set

$$\begin{aligned} B(u_0) = \{ u \in {\mathcal {A}}_{2,\infty }^k(r,T) :\Vert u\Vert _{{\mathcal {A}}^k(r,T)} \le 2C\Vert u_0\Vert _{A_2^k(r)}\}. \end{aligned}$$

In order to do so, we recall (4.16) and (4.17), and notice that those are still valid in case $d \ge 2$ and $j, k_1,k_2,k_3$ are multiindices of integers. Instead of employing Strichartz estimates, we use the fact that, for $f,g,h \in {\mathcal {S}}(\mathbb {R}^d),$ we have the following trilinear estimate

$$\begin{aligned} \Vert f g h \Vert _{2,k} \lesssim \Vert f\Vert _{2,k} \Vert g\Vert _{\infty } \Vert h\Vert _{\infty } + \Vert f\Vert _{\infty } \Vert g\Vert _{2,k} \Vert h\Vert _{\infty } + \Vert f\Vert _{\infty } \Vert g\Vert _{\infty } \Vert h\Vert _{2,k}. \end{aligned}$$

Thus, we may estimate

$$\begin{aligned} \Vert \Gamma ^j(\Phi (u))(t)\Vert _{2,k}&\le C \Vert x^j u_0\Vert _2 \\&+ \sum _{k_1 + k_2 + k_3 = j} \frac{j!}{(k_1)! (k_2)! (k_3)!} \int _0^t \Vert \Gamma ^{k_1} u\Vert _{\infty } \cdot \Vert \Gamma ^{k_2} u \Vert _{\infty } \cdot \Vert \Gamma ^{k_3} u\Vert _{2,k} \, d s \\&+ \text {analogous terms}. \end{aligned}$$

By Sobolev’s inequality, we are able to bound each $L^{\infty }$ norm above by the $(2,k)-$norms, as long as $k > \frac{d}{2}.$ Thus, we have

$$\begin{aligned}&\sup _{t \in [0,T]} \Vert \Gamma ^j (\Phi (u))\Vert _{2,k} \lesssim C \Vert x^j u_0\Vert _2 \\&+ T \sum _{k_1 + k_2 + k_3 = j} \frac{j!}{(k_1)!(k_2)!(k_3)!} \left( \sup _{t \in [0,T]} \Vert \Gamma ^{k_1} u\Vert _{2,k} \right) \cdot \left( \sup _{t \in [0,T]} \Vert \Gamma ^{k_2} u \Vert _{2,k}\right) \cdot \left( \sup _{t \in [0,T]} \Vert \Gamma ^{k_3} u\Vert _{2,k} \right) . \end{aligned}$$

the last factors by $r^{|j|}/j!$ and summing up in $j \in (\mathbb {Z}_{\ge 0})^d,$ we obtain

$$\begin{aligned} \Vert \Phi (u)\Vert _{{\mathcal {A}}^k(r,T)} \lesssim \Vert u_0\Vert _{A^k(r)}+ T\Vert u\Vert _{{\mathcal {A}}^k(r,T)}^3. \end{aligned}$$

In the same way, for $u,v \in B(u_0)$ we obtain

$$\begin{aligned} \Vert \Phi (u) - \Phi (v)\Vert _{{\mathcal {A}}^k(r,T)} \le C T (\Vert v\Vert _{{\mathcal {A}}^k(r,T)}^2 + \Vert u\Vert _{{\mathcal {A}}^k(r,T)}^2) \Vert u-v\Vert _{{\mathcal {A}}^k(r,T)}. \end{aligned}$$

Thus, if $T \le \frac{1}{C'\Vert u_0\Vert _{A_2^k(r)}^2},$ for $C'$ sufficiently large, $\Phi$ becomes a contraction in $B(u_0),$ and thus we are able to conclude the claims, similarly as in the proof of Lemma 4.4. $\square$

From these results, we use the following argument: as

$$\begin{aligned} \Vert u\Vert _{{\mathcal {A}}_{2,\infty }^k(r,T)} = \sum _{j \in (\mathbb {Z}_{\ge 0})^d} \frac{r^{|j|}}{j!} \left( \sup _{t \in [0,T]} \Vert \Gamma ^j u\Vert _{2,k} \right) , \end{aligned}$$

we get in particular that

$$\begin{aligned} \sum _{j \in (\mathbb {Z}_{\ge 0})^d} \frac{r^{|j|}}{j!} \Vert \Gamma ^j u\Vert _{2}&=\sum _{j \in (\mathbb {Z}_{\ge 0})^d} \frac{(|t|r)^{|j|}}{j!} \Vert \partial _x^j(e^{i|x|^2/4t} u)\Vert _2 \\&= \sum _{j \in (\mathbb {Z}_{\ge 0})^d} \frac{(2\pi r|t|)^{|j|}}{j!} \Vert x^j {\mathcal {F}}(e^{i|x|^2/4t} u(t))\Vert _2 \end{aligned}$$

is uniformly bounded in $t \in [0,T]$. Just as noted before, one may prove that this directly implies that

$$\begin{aligned} \Vert e^{2 \pi r|t|(|x_1| + \cdots + |x_d|)} {\mathcal {F}}(e^{i|x|^2/4t} u(t))\Vert _2 \text { is uniformly bounded in } t \in [0,T]. \end{aligned}$$

But a simple Fourier analysis argument shows that, if the equation above holds, then we have

4.18 $$\begin{aligned} e^{i|z|^2/4t} u(t) \text { is analytic in } S(r|t|), \end{aligned}$$

where we use the notation $|z|^2 = z_1^2 + \cdots + z_k^2,$ and $S(a) = \{ z \in \mathbb {C}^d; :|\text {Im}(z_j)| < a, \forall j \in \{1,\dots ,d\} \}.$ In particular, it can also be shown that $e^{i|z|^2/4t} u(t)$ is analytic and bounded in *any* strip $S(r'),$ where $r' < r |t|.$

One may wonder whether the lemmata 4.4 and 4.5 can be improved, in the sense that, if $u_0$ is analytic in a strip *S*(*r*) and possesses exponential decay, then a solution *u*(*t*) to the IVP (2.5) is analytic in a slightly larger strip $S(r+\varepsilon (t)),$ for some (positive) $\varepsilon (t)$ depending on time, and some—possibly large—time $t >0.$

For the sake of comparison, let us first analyse the case of the free Schrödinger equation. If *v*(*x*, *t*) is a solution to

4.19 $$\begin{aligned} {\left\{ \begin{array}{ll} i \partial _t v + \partial _x^2 v = 0, \\ v(x,0) = u_0(x), \\ \end{array}\right. } \end{aligned}$$

then we may write $v(x,t) = \frac{e^{ix^2/4t}}{(4\pi i t)^{1/2}} \widehat{(e^{i(\cdot )^2/4t} u_0)} \left( \frac{x}{4\pi t}\right) .$ If $|u_0(x)| \le C e^{-A|x|},$ then an argument with the Fourier transform and Morera’s theorem shows that *v*(*x*, *t*) is analytic in a strip $S(2 \pi ^2 t A).$ If we suppose, for instance, that $u_0 \in L^2(S(r), z \, dz),$ then Lemma 1 in Hayashi–Saitoh [11] shows that, in fact, $\widehat{u_0} \in L^2(e^{4\pi r|z|} \, dz),$ which directly implies that *v*(*x*, *t*) is analytic in *S*(*r*),  for any $t >0.$ Thus, decay and (a certain level of) analyticity are seen to preserve and, for large times, improve how analytic the solution to (4.19) is.

On the other hand, analyticity in such a strip is seen to be sharp, even if $u_0$ is analytic. Indeed, letting $f_0(x) = \frac{2}{e^{\pi x} + e^{-\pi x}},$ it is a classical result that $\widehat{f_0}(\xi ) = f_0(\xi ).$ Let then *v*(*x*, *t*) be a solution to (4.19) with initial data $u_0(x) = e^{-ix^2/4t_0} f_0(x).$ For time $t = t_0,$ we know that the solution may be written as

$$\begin{aligned} v(x,t_0) = \frac{e^{ix^2/4t_0}}{(4\pi i t_0)^{1/2}} f_0(x/(4\pi t_0)), \end{aligned}$$

which is seen to be analytic in $S(2 \pi t_0)$ but not in a larger strip.

In the case of the cubic NLS, we see that this long-time analytic improvement is, in general, *false*. Indeed, one easily sees that

$$\begin{aligned} u(x,t) = e^{i \pi ^2 t} \sqrt{2} \pi f_0(x) \end{aligned}$$

is a solution to (2.5). As a solitary wave, it preserves, for any time $t \in \mathbb {R},$ the same properties of the initial datum $\sqrt{2} \pi f_0(x).$ In particular, $u(\cdot , t)$ decays as $e^{-\pi |x|}$ for any time, and is seen to be analytic in the strip *S*(1/2),  with poles at $\pm \frac{i}{2}.$ This shows, in particular, that the nonlinearity induces a different long-time analytic behaviour in the dynamic of the equation, and that in general analyticity cannot be improved for the cubic NLS even if there is some exponential gain.

Finally, we state a result on how zeros of a function *f*, where *f* is assumed to be analytic and bounded in a strip, may behave.

#### Lemma 4.6

Let *f* be analytic and bounded in the strip *S*(*r*). Let $\{z_n\}_{n \ge 0} = \{x_n + i y_n\}_{n \ge 0}$ denote an enumeration of the zeros of *f*,  accounting for multiplicity. Then the following condition must be satisfied:

$$\begin{aligned} \sum _{n \ge 0} \frac{e^{\pi x_n/(2r)} \cos (\pi y_n/(2r))}{((e^{\pi x_n/r} + 1)^2 - 4e^{\pi x_n/r} \cos ^2(\pi y_n/(2r)))^2)^{1/2}} < + \infty . \end{aligned}$$

#### Proof

We start by noticing that the map $\varphi (z) = \frac{e^{\frac{z\pi }{2r}} - 1}{e^{\frac{z \pi }{2r}} + 1}$ maps the strip *S*(*r*) biholomorphically into the unit disc ${\mathbb {D}} = \{ z \in \mathbb {C}:|z| < 1\},$ and moreover it takes the closure $\overline{S(r)}$ of the strip into the closure of the unit disc $\overline{{\mathbb {D}}}.$

Thus, the function $f \circ \varphi ^{-1} : {\mathbb {D}} \rightarrow \mathbb {C}$ is holomorphic inside the unit disc ${\mathbb {D}},$ and bounded up to the boundary $\overline{{\mathbb {D}}}.$ We now use a theorem by Szegő (see also [6, Chapter 2, Theorem 2.1]): if $g : {\mathbb {D}} \rightarrow \mathbb {C}$ is a non-zero bounded analytic function, then the zeros $\{w_n\}_{n \ge 0},$ enumerated accounting for multiplicity, satisfy

$$\begin{aligned} \sum _{n \ge 0} (1-|w_n|) < + \infty . \end{aligned}$$

If $f \not \equiv 0,$ then $f \circ \varphi ^{-1}$ satisfies the conditions of this result. As we know that $w_n = \frac{e^{\frac{z_n \pi }{2r}} - 1}{e^{\frac{z_n \pi }{2r}} +1}$ are the zeros of $f \circ \varphi ^{-1},$ we must have then

$$\begin{aligned} \sum _{n \ge 0} \left( 1 - \left| \frac{e^{\frac{z_n \pi }{2r}} - 1}{e^{\frac{z_n \pi }{2r}} + 1}\right| \right) < + \infty . \end{aligned}$$

But using that

$$\begin{aligned} \left| \frac{e^{\frac{z_n \pi }{2r}} - 1}{e^{\frac{z_n \pi }{2r}} + 1}\right| = \left( 1 - 4 \frac{e^{x_n \pi /(2r)} \cos (\pi y_n/(2r))}{e^{\pi x_n/r} + 2 e^{\pi x_n/(2r)} \cos (\pi y_n/(2r)) + 1} \right) ^{1/2}, \end{aligned}$$

and estimating $1 - (1-\alpha )^{1/2} \ge \frac{\alpha }{4(1-\alpha )^{1/2}}$ for $\alpha > 0$ small, we obtain that a necessary condition that the zeros of *f* must satisfy is

$$\begin{aligned} \sum _{n \ge 0} \frac{e^{\pi x_n/(2r)} \cos (\pi y_n/(2r))}{((e^{\pi x_n/r} + 1)^2 - 4(e^{\pi x_n/(2r)} \cos (\pi y_n/(2r)))^2)^{1/2}} < + \infty . \end{aligned}$$

This finishes the proof. $\square$

Notice that, if the sequence of zeros satisfies $y_n = 0,$ then this result has a much more pleasant formulation. Indeed, we may rewrite the condition then as

4.20 $$\begin{aligned} \sum _{n \ge 0} \frac{e^{\pi x_n/(2r)}}{e^{\pi x_n /r} - 1} = \sum _{n \ge 0} \frac{1}{e^{\pi x_n/(2r)} - e^{-\pi x_n/(2r)}}< + \infty . \end{aligned}$$

This is, in fact, the form of this result that shall be useful to us when dealing with some specific sequences, such as the ones described in our main results.

### Decay from Zeros

Finally, we address the question of how to obtain decay for a solution to (2.5) given the locations of its zeros.

We start with the one-dimensional case, where we have the following stronger result:

#### Lemma 4.7

Let $u \in C([0,T] :H^1(\mathbb {R}))$ be a strong solution to (2.5). Suppose that

$$\begin{aligned} u_0(\pm c_1 \log (1+n)^{\alpha }) = u(\pm c_2 \log (1+n)^{\alpha }, T) = 0, \, \forall \, n \ge 0, \end{aligned}$$

and some $\alpha \in (0,1].$ Then, for each $N > 0,$ there is $C_N > 0$ so that

$$\begin{aligned} |u_0(x)| + |u(x,T)| \le C_N e^{-N|x|}. \end{aligned}$$

#### Proof

We start by noticing that, since $u \in C([0,T] :H^1(\mathbb {R})),$ then we may use a simple comparison for both times $t=0,T,$ using the fundamental theorem of calculus. Indeed, for $t=0$ for instance, we have,

$$\begin{aligned} |u_0(x)| =&|u_0(x) - u_0(c_1 \log (1+n)^{\alpha })| \le \int _x^{c_1 \log (1+n)^{\alpha }} |u_0'(t)| \, dt \\&\le |c_1 \log (1+n)^{\alpha } - c_1 \log (n)^{\alpha }|^{1/2} \Vert u_0 ' \Vert _2 \le 2 c_1^{1/2} \frac{\Vert u_0'\Vert _2}{(n+1)^{1/2}}\\&\le 4 c_1 e^{-|x|/(2c_1)} \Vert u_0 '\Vert _2. \end{aligned}$$

Here, we have taken $x \in (c_1 \log (n), c_1 \log (1+n)),$ without loss of generality, with $n \in \mathbb {N}$ sufficiently large. In particular, this shows exponential decay for the initial data and, by applying the same procedure to the data at time $t=T,$ we have

$$\begin{aligned} |u(x,T)| \le 4 c_2^{1/2} e^{-|x|/(2c_2)} \Vert \partial _x u(T) \Vert _2. \end{aligned}$$

By Lemma 4.1, this readily implies that $u \in C([0,T] :H^k \cap L^2(x^{2k})),$ for any $k \in \mathbb {N}.$ In particular, we know that $\partial _x^k u_0 , \partial _x^k u(T)\in L^2(\mathbb {R}), \, \forall k \in \mathbb {N}.$

Before moving on, we divert our attention for a second towards the following property, which we state as a lemma:

#### Lemma 4.8

Let $f: \mathbb {R}\rightarrow \mathbb {C}$ be a (smooth) function with a sequence of zeros at $\{x_n\}_{n \ge 0}.$ Write $f = u + i v.$ We may find, for each $k \ge 0,$ sequences $\{a^{(k)}_n\}_{n \ge k}, \{b^{(k)}_n\}_{n \ge k}$ so that the following conditions are met:

1. $$\{a^{(0)}_n\}_{n \ge 0} = \{ b^{(0)}_n \}_{n \ge 0} = \{x_n\}_{n \ge 0};$$
2. For each $n \ge k,$ we have $a^{(k)}_{n} \in (a^{(k-1)}_{n-1}, a^{(k-1)}_{n})$ and $b^{(k)}_{n} \in (b^{(k-1)}_{n-1}, b^{(k-1)}_{n});$
3. For each $n \ge k,$ we have $\partial _x^k u(a^{(k)}_n) = \partial _x^k v( b^{(k)}_n ) = 0.$

The proof of this result follows by a simple inductive argument, using the mean value theorem, so we omit it. Notice that, in particular, if we start off with $x_n = c \log (1+n)^{\alpha },$ with $c \in \{c_1,c_2\},$ we may take *f* to be either $u_0$ or *u*(*T*). This implies that there are, for each $k \ge 0,$ sequences

$$\begin{aligned} \{a^{(k)}_n\}_{n \ge k}, \{b^{(k)}_n\}_{n \ge k}, \{ c^{(k)}_n\}_{n \ge k}, \, \{d^{(k)}_n\}_{n \ge k}, \end{aligned}$$

so that

$$\begin{aligned} \partial _x^k \text {Re} (u_0)(a^{(k)}_n)= & {} \partial _x^k \text {Im}(u_0)(b^{(k)}_n) \\= & {} \partial _x^k \text {Re}(u(T)) (c^{(k)}_n) = \partial _x^k \text {Im}(u(T))(d^{(k)}_n) = 0, \, \forall \, n \ge k, \, k \ge 0. \end{aligned}$$

In particular, from the inductive construction of these sequences, we see that $\eta ^{(k)}_n \in (c \log (n-k), c \log (1+n)),$ where $c \in \{c_1,c_2\}$ and $\eta \in \{a,b,c,d\}.$

Now, in order to conclude the argument, notice that, by applying the argument, which we used to deduce decay above, to the $k-$th derivatives of the real and imaginary parts of $u_0$ and *u*(*T*),  we have that, for some $C(k) > 0,$

$$\begin{aligned} |\partial _x^k \text {Re} (u_0)(x)| + |\partial _x^k \text {Im}(u_0)(x)| \le C(k) c_1^{1/2} e^{-|x|/(2c_1)} \Vert \partial _x^{k+1} (u_0) \Vert _2, \end{aligned}$$

and analogously,

$$\begin{aligned} |\partial _x^k \text {Re}(u(T))(x)| + | \partial _x^k \text {Im}(u(T))(x)| \le C(k) c_2^{1/2} e^{-|x|/(2c_2)} \Vert \partial _x^{k+1} (u(T)) \Vert _2. \end{aligned}$$

Suppose then $y \in (a^{(k)}_n,a^{(k)}_{n+1}).$ Then

$$\begin{aligned} |\partial _x^{k-1} \text {Re}(u_0)(y)| \le \int _y^{a^{(k)}_{n+1}} |\partial _x^k \text {Re}(u_0)(x)| \, dx \le {\tilde{C}}(k) c_1 e^{-|y|/c_1} \Vert \partial _x^{k+1} (u_0)\Vert _2. \end{aligned}$$

Analogous estimates hold for $\partial _x^{k-1} \text {Im}(u_0), \partial _x^{k-1} \text {Re}(u(T)), \partial _x^{k-1} \text {Im}(u(T)),$ and we may use this process of improving backwards in order to obtain that, for each $k > 0,$

$$\begin{aligned} |u_0(x)| \le&|\text {Re}(u_0)(x)| + |\text {Im}(u_0)(x)| \le C'(k) c_1^{k/2} e^{-k|x|/(2c_1)} \Vert \partial _x^{k+1} (u_0)\Vert _2,\\ |u(x,T)| \le&|\text {Re}(u(T))(x)| + |\text {Im}(u(T))(x)| \le C'(k) c_2^{k/2} e^{-k|x|/(2c_2)} \Vert \partial _x^{k+1} (u(T)) \Vert _2. \end{aligned}$$

By making $k > 0$ sufficiently large, we conclude the assertion, as desired. $\square$

In higher dimensions, we have a slightly worse version of the result above.

#### Lemma 4.9

Let $u \in C([0,T] :H^s(\mathbb {R}^d))$, $s > \frac{d}{2}-1$ be a strong solution to (2.5), where $d \ge 2.$ If $d=2$ or $d=3$, assume additionally that $s\ge 1$. Suppose that

$$\begin{aligned} u_0(\pm c_1 \log (1+n)^{\alpha }{\mathbb {S}}^{d-1}) = u(\pm c_2 \log (1+n)^{\alpha } {\mathbb {S}}^{d-1}, T) = 0, \, \forall \, n \ge 0, \end{aligned}$$

where the identity is to be interpreted in the sense of Sobolev traces, for some $\alpha \in (0,1].$ Then, for any $N \ge 0$ fixed real number, there is a positive constant $c_N > 0$ so that

$$\begin{aligned} |\partial ^{\alpha } u_0(x)| + |\partial ^{\alpha }u(x,T)| \lesssim _{N,u_0,u(T)} e^{-c_N|x|}, \end{aligned}$$

for all multiindices $\alpha$ so that $|\alpha | \le N.$

#### Proof

The proof consists of two steps, one where one obtains exponential decay for both $u_0, u(T)$, and an interpolation argument in order to obtain exponential decay for higher derivatives.

**Step 1:** $u_0, u(T)$ **are bounded by** $e^{-c_0|x|},$ **where** $c_0 >0$ **depends on the dimension and on the parameters** $c_1,c_2.$ For this step, the idea is to use Poincaré’s inequality between spheres where zeros are located.

Indeed, for that we will need the following proposition:

#### Proposition 4.10

Let $C(\Omega )$ denote the best constant in the Poincaré inequality

$$\begin{aligned} \Vert u\Vert _{L^2(\Omega )} \le C(\Omega ) \Vert \nabla u\Vert _{L^2(\Omega )} \end{aligned}$$

for $u\in H_0^1(\Omega )$. If $\Omega = B_R \setminus B_r, \, r \in (0,R),$ then

$$\begin{aligned} C(\Omega ) \lesssim _d (R^d - r^d)^{2/d}. \end{aligned}$$

#### Proof of the Proposition

It is a well-known fact that the best constant in Poincaré’s inequality in a domain $\Omega$ is $1/\lambda _1,$ where $\lambda _1$ is the first eigenvalue of the Laplacian with Dirichlet boundary conditions on $\Omega .$ Thus, we only need to estimate this eigenvalue.

In order to do that, we use the Faber–Krahn inequality, which says that

$$\begin{aligned} \lambda _1(\Omega ) \ge \lambda _1 (B), \end{aligned}$$

where *B* is a ball with $|B| = |\Omega |.$ Thus, taking into account the eigenvalue of the Dirichlet problem for the ball, we have

$$\begin{aligned} \lambda _1(\Omega ) \ge c_d \cdot \left( \frac{1}{|\Omega |} \right) ^{2/d}, \end{aligned}$$

where $c_d = \pi \cdot \Gamma (d/2 + 1)^{2/d} \cdot j_{d/2 - 1},$ where $j_{m,1}$ denotes the first positive zero of the Bessel function $J_m.$ Thus, we obtain the control

$$\begin{aligned} C(\Omega ) \le \frac{1}{c_d} |\Omega |^{2/d} = {\tilde{c}}_d (R^d - r^d)^{2/d}. \end{aligned}$$

This ends the proof of the proposition. $\square$

With this proposition, we use the Poincaré inequality above with $u_0, u(T)$ in each of the annuli $A_{i,n} ;= B_{c_i \log (1+n)^{\alpha }} \setminus B_{c_i \log (n)^{\alpha } }, n \ge 1.$ We obtain that

$$\begin{aligned} \Vert u_0\Vert _{L^2(A_{1,n})}&+ \Vert u(T)\Vert _{L^2(A_{2,n})} \\&\lesssim \log (n+1)^{2\alpha \cdot \frac{d-1}{d}} (\log (n+1)^{\alpha } - \log (n)^{\alpha })^{2/d} \left( \Vert \nabla u_0\Vert _{L^2(A_{1,n})} + \Vert \nabla u(T)\Vert _{L^2(A_{2,n})} \right) \\&\lesssim \frac{\log (n+1)^{2\alpha \cdot \frac{d-1}{d}}}{(n+1)^{2/d}} \left( \Vert \nabla u_0\Vert _{L^2(A_{1,n})} + \Vert \nabla u(T)\Vert _{L^2(A_{2,n})} \right) . \end{aligned}$$

Reordering terms, we obtain that

$$\begin{aligned} \left\| \frac{e^{2|x|/(d c_1)}}{(1+|x|)^2} u_0 \right\| _{L^2(A_{1,n})}&+ \left\| \frac{e^{2|x|/(d c_2)}}{(1+|x|)^2} u(T) \right\| _{L^2(A_{2,n})}\\&\lesssim \Vert \nabla u_0\Vert _{L^2(A_{1,n})} + \Vert \nabla u(T)\Vert _{L^2(A_{2,n})}. \end{aligned}$$

Summing over $n \ge 0,$ we obtain that

$$\begin{aligned} \left\| \frac{e^{2|x|/(d c_1)}}{(1+|x|)^2} u_0 \right\| _2+ \left\| \frac{e^{2|x|/(d c_2)}}{(1+|x|)^2} u(T) \right\| _2 \lesssim \Vert \nabla u_0\Vert _{L^2} + \Vert \nabla u(T)\Vert _{L^2}. \end{aligned}$$

We shall now use the following proposition, whose proof is inspired in that of [23, Prop 3.1]:

#### Proposition 4.11

Let $f \in L^2(e^{2a|x|}dx), \nabla f \in L^{\infty }.$ Then $|f(x)| \le C e^{-\frac{a}{d+2}|x|}, \, \forall x \in \mathbb {R}^d$, where *C* depends on $\Vert f\Vert _{L^\infty }, \Vert \nabla f \Vert _{L^\infty }, \Vert f\Vert _{L^2 (e^{a|x|} dx) }$.

#### Proof

Without loss of generality, *f* is not identically zero. As $\nabla f \in L^\infty$, we have that *f* is absolutely continuous so it suffices to consider large |*x*|. A standard argument shows that $\nabla f \in L^\infty$ and $f\in L^2$ imply that $f(x) \rightarrow 0$ as $x\rightarrow \infty$. In particular, $f\in L^\infty$. Now, for each $x \in \mathbb {R}^d,$ fix a ball $B_x$ centered at *x*,  with radius $r_x>0$ to be determined in a while. Then, for any $y \in B_x,$ we have

$$\begin{aligned} |f(x) - f(y)| \le \left( \sup _{z \in B_x} |\nabla f(z)| \right) |x-y| \le 2r_x \Vert \nabla f\Vert _{\infty }. \end{aligned}$$

We then pick $r_x = \frac{|f(x)|}{4\Vert \nabla f\Vert _{\infty }},$ and thus $|f(y)| \ge |f(x)|/2.$ Note also that $\sup _x r_x <\infty$ as $f\in L^\infty$. Moreover, if |*x*| is sufficiently large, then triangle’s inequality shows that $|y| \ge |x|/2, \, \forall y \in B_x.$ Therefore, for such *x*, 

$$\begin{aligned} \Vert f e^{a|\cdot |}\Vert _2^2 \ge \int _{B_x}| e^{a|y|}f(y) |^2 \, d y \gtrsim r_x^d e^{a|x|} |f(x)|^2 \gtrsim _{f} |f(x)|^{d+2} e^{{a}|x|}. \end{aligned}$$

This readily implies that $|f(x)| \lesssim _f e^{-\frac{a}{(d+2)} |x|}, \forall |x| \gg 1.$ This finishes the proof. $\square$

We now employ Lemma 4.2 for $u_0, u(T).$ This shows, in particular, that $\nabla u_0, \nabla u(T) \in L^{\infty }.$ By Proposition 4.11, we are able to finish **Step 1.**

**Step 2: Propagating decay for derivatives.** For this step, we use an inductive argument on the number $N = |\alpha |$. In particular, if $N = 0,$ we are done by **Step 1.**

Suppose our result holds for $N \in \mathbb {N}$ fixed. By Lemma 4.2, we are able to deduce that derivatives of *all* orders are bounded. We use this fact, together with a Taylor expansion of order one in order to conclude.

Indeed, fixing $\alpha$ a multiindex so that $|\alpha | = N+1,$ let $j \in \{1,\dots ,d\}$ be so that $\alpha _j > 0.$ In particular, we may perform a Taylor expansion of order one of the function $\partial _x^{\alpha -e_j} u_0(x+te_j).$ In particular, we know that, since the derivative $\partial _x^{\alpha +e_j}u_0$ belongs to $L^{\infty }(\mathbb {R}^d),$

$$\begin{aligned} \left| \partial _x^{\alpha -e_j} u_0(x+te_j) - \partial _x^{\alpha -e_j}u_0(x) - t \partial _x^{\alpha } u_0(x) \right| \lesssim _{\Vert \partial _x^{\alpha + e_j} u_0 \Vert _{L^\infty }} t^2. \end{aligned}$$

Thus, reordering terms,

$$\begin{aligned} |\partial _x^{\alpha }u_0(x)|&\lesssim _{u_0} t^{-1}\left( | \partial _x^{\alpha -e_j} u_0(x+te_j)| + |\partial _x^{\alpha -e_j}u_0(x)| \right) + t \\&\lesssim _{u_0} t^{-1} \left( e^{-c_N|x+te_j|} + e^{-c_N|x|} \right) + t, \end{aligned}$$

where we used the induction hypothesis in the last line. In particular, we may choose $t = e^{-\frac{c_N}{2} |x|}$ above. Routine computations then imply

$$\begin{aligned} |\partial _x^{\alpha }u_0(x)| \lesssim _N e^{-\frac{c_N}{2}|x|}. \end{aligned}$$

This closes the inductive argument, and we are done. $\square$

## Proof of the One-dimensional Results

### Proof of the Unique Continuation Results

#### Proof of Theorem 1

By using the invariance of the Schrödinger equation and the associated properties of the potential (see, for instance, [5, p. 180]), we may suppose that $T=1.$

We now use the Duhamel formula for representing the solution as

5.1 $$\begin{aligned} u(x,t) = e^{it\partial _x^2}u_0(x) + \int _0^t e^{(t-\tau )i\partial _x^2} (V(\tau ) u(\tau ))(x) \, d\tau , \, \forall \, t \in [0,1]. \end{aligned}$$

**Step 1**: $u_0,u(1)$ **are** $\frac{1}{2}$-**Hölder continuous.** Using (5.1) we estimate for some $\theta >0$

$$\begin{aligned} |u(x,1)-u(y,1)|&\le |e^{i\partial _x^2} u_0 (x) - e^{i\partial _x^2} u_0 (y)| \\&+ \int _0^{1-\theta } |e^{(1-s)\partial _x^2} ( V(s) u(s))(x) - e^{(1-s)\partial _x^2} ( V(s) u(s))(y)| ds\\&+ \int ^1_{1-\theta } |e^{(1-s)\partial _x^2} ( V(s) u(s))(x) - e^{(1-s)\partial _x^2} ( V(s) u(s))(y)| ds. \end{aligned}$$

For the first term we use the first bound in Lemma 3.1 to obtain

$$\begin{aligned} |e^{i\partial _x^2} u_0 (x) - e^{i\partial _x^2} u_0 (y)|\lesssim \max ({|x|,|y|}) \Vert (1+|z|) u_0(z) \Vert _{L^1} |x-y|. \end{aligned}$$

For the second term we again use the first bound in Lemma 3.1 and obtain

$$\begin{aligned}&\int _0^{1-\theta } |e^{(1-s)\partial _x^2} ( V(s) u(s))(x) - e^{(1-s)\partial _x^2} ( V(s) u(s))(y)| ds\\&\lesssim \max (|x|,|y|)|x-y| \int _0^{1-\theta } \frac{\Vert (1+|z|) V(z,s) u(z,s)\Vert _{L^1} }{(1-s)^{\frac{3}{2}}} d s \\&\lesssim \max (|x|,|y|)|x-y| \theta ^{-\frac{1}{2}} \Vert V u \Vert _{L^\infty _{[0.1]} L^1((1+|z|) dz)}. \end{aligned}$$

For the third term we use the second bound in Lemma 3.1 to obtain

$$\begin{aligned} \int ^1_{1-\theta } |e^{(1-s)\partial _x^2} ( V(s) u(s))(x) - e^{(1-s)\partial _x^2} ( V(s) u(s))(y)| ds&\lesssim \int ^1_{1-\theta } \frac{ \Vert V(s) u(s)\Vert _{L^1} }{(1-s)^{\frac{1}{2}} } ds \\&\lesssim \theta ^{\frac{1}{2}} \Vert V u \Vert _{L^\infty _{[0,1]} L^1}. \end{aligned}$$

We first observe that

$$\begin{aligned} \Vert V u \Vert _{L^\infty _{[0,1]} L^1} \lesssim \Vert V\Vert _{L^\infty _{[0,1]} L^2} \Vert u\Vert _{L^\infty _{[0,1]} L^2} < \infty , \end{aligned}$$

as well as

$$\begin{aligned}\Vert V u \Vert _{L^1_{[0,1]} L^1((1+|z|)dz) } \lesssim \Vert V\Vert _{L^\infty _{[0,1]} L^2((1+|z|^2) d z) } \Vert u\Vert _{L^\infty _{[0,1]} L^2} < \infty \end{aligned}$$

in view of the assumptions of Theorem 1. Then, choosing $\theta = |x-y| \max (|x|,|y|)$ shows that

5.2 $$\begin{aligned} |u(x,1)-u(y,1)|\le C(\Vert V\Vert _{L^\infty _{[0,1]} L^2((1+|z|^2) d z )}, \Vert u\Vert _{L^\infty _{[0,1]} L^2}) |x-y|^{\frac{1}{2}} \max (|x|,|y|)^{\frac{1}{2}} \end{aligned}$$

which shows that $u(\cdot , 1)$ is $\frac{1}{2}$-Hölder continuous. Evolving backwards, we analogously obtain $\frac{1}{2}$-Hölder continuity for $u_0$.

**Step 2: Decay estimate from the zeros.** With the $C^{\frac{1}{2}}$-continuity at hand, we will make use of the pointwise assumptions. The idea is then to compare the solution at two different points for times $t=0,1.$ In order to do so, we argue similarly to **Step 1** and make use of Lemma 3.1. Indeed, suppose that $|u(\pm c_1 \log (1+n)^{\alpha },1)| \le n^{-\delta },$ for some $\delta > 0.$ Suppose that

$$\begin{aligned} x \in ( c_1 \log (1+n)^{\alpha }, c_1 \log (2+n)^{\alpha }).\end{aligned}$$

Then, as before, using the Duhamel formula for $t=1,$

5.3 $$\begin{aligned} |u(x,1)| \le&|u(x,1) - u(c_1 \log (1+n)^{\alpha },1)| + n^{-\delta } \nonumber \\&\le |e^{i\partial _x^2}u_0(x) - e^{i\partial _x^2}u_0(c_1 \log (1+n)^{\alpha })| \nonumber \\&+ \int _0^{1-\theta } |e^{(1-s)i\partial _x^2} (V(s) u(s))(x) - e^{(1-s)i\partial _x^2} (V(s) u(s))(c_1 \log (1+n)^{\alpha })| \, ds \nonumber \\&+ \int _{1-\theta }^1 |e^{(1-s)i\partial _x^2} (V(s) u(s))(x) - e^{(1-s)i\partial _x^2} (V(s) u(s))(c_1 \log (1+n)^{\alpha })| \, ds + n^{-\delta }. \end{aligned}$$

We may use the first bound in Lemma 3.1 directly in the first term in the sum above. As $x \in ( c_1 \log (1+n)^{\alpha }, c_1 \log (2+n)^{\alpha }),$ then $|c_1 \log (1+n)^{\alpha }| \le 2|x|$ if $n \ge 1.$ This implies that

$$\begin{aligned} |e^{i\partial _x^2}u_0(x) - e^{i\partial _x^2}u_0(c_1 \log (1+n)^{\alpha })|&\le C|x||x - c_1 \log (1+n)^{\alpha }| \Vert (1+|z|)u_0(z)\Vert _1 \\&\le C\cdot c_1 |x| |\log (2+n)^{\alpha } - \log (1+n)^{\alpha }| | \Vert (1+|z|)u_0(z)\Vert _1 \\&\le {\tilde{C}} |x| e^{-\frac{1}{2} |x|^{1/\alpha }} \Vert (1+|z|)u_0(z)\Vert _1. \end{aligned}$$

Similarly to **Step 1** we estimate

$$\begin{aligned} \int _0^{1-\theta }&|e^{(1-s)i\partial _x^2} (V(s) u(s))(x) - e^{(1-s)i\partial _x^2} (V(s) u(s))(c_1 \log (1+n)^{\alpha })| \, ds \\&+ \int _{1-\theta }^1 |e^{(1-s)i\partial _x^2} (V(s) u(s))(x) - e^{(1-s)i\partial _x^2} (V(s) u(s))(c_1 \log (1+n)^{\alpha })| \, ds \\ \le&C |x||x - c_1 \log (1+n)^{\alpha }| \int _0^{1-\theta } \frac{\Vert (1+|z|) V(z,s) u(z,s)\Vert _1}{(1-s)^{3/2}} \, ds \\&+ C \int _{1-\theta }^1 \frac{\Vert V(z,s)u(z,s)\Vert _1}{(1-s)^{1/2}} \, ds \le C \theta ^{-1/2} |x|e^{-\frac{1}{2}|x|^{1/\alpha }} \Vert Vu\Vert _{L^\infty _{[0,1]} L^1((1+|z|)dz)} \\&+ C \theta ^{1/2} \Vert Vu\Vert _{L^{\infty }_{[0,1]} L^1(\mathbb {R})}. \end{aligned}$$

We want to make both estimates on the right-hand side above compatible, so we take $\theta = |x|e^{-\frac{1}{2}|x|^{1/\alpha }} \frac{\Vert Vu\Vert _{L^\infty _{[0,1]} L^1((1+|z|)dz)}}{\Vert Vu\Vert _{L^{\infty }_{[0,1]} L^1(\mathbb {R})}}.$ This yields that the second and third terms in (5.3) are bounded by

$$\begin{aligned} C|x|^{1/2} e^{-\frac{1}{4} |x|^{1/\alpha }} \Vert Vu\Vert _{L^\infty _{[0,1]} L^1((1+|z|)dz)}^{1/2} \Vert Vu\Vert _{L^{\infty }_{[0,1]} L^1(\mathbb {R})}^{1/2}. \end{aligned}$$

On the other hand, by a simple use of Cauchy–Schwarz, this last term is bounded by

$$\begin{aligned} C|x|^{1/2} e^{-\frac{1}{4} |x|^{1/\alpha }} \Vert u\Vert _{L^{\infty }_{[0,1]} L^2(\mathbb {R})} \Vert V\Vert _{L^{\infty }_{[0,1]} L^2((1+|z|)^2 dz)}. \end{aligned}$$

Finally, as $|x| \le c_1 \log (2+n)^{\alpha }$ and

$$\begin{aligned}(2+n)^{-\delta } = e^{- \delta \log (n+2)} = e^{- \delta \frac{(c_1 \log (n+2)^{\alpha })^{1/\alpha }}{c_1^{1/\alpha }}} \le e^{-\frac{\delta }{c_1^{1/\alpha }} |x|^{1/\alpha }},\end{aligned}$$

we may estimate then

5.4 $$\begin{aligned}&|u(x,1)| \nonumber \\&\le C|x| e^{-\omega |x|^{1/\alpha }} \left( 1 + \Vert (1+|z|)u_0\Vert _1 + \Vert u\Vert _{L^{\infty }_{[0,1]} L^2(\mathbb {R})} \Vert V\Vert _{L^{\infty }_{[0,1]} L^2((1+|z|)^2 dz)} \right) ,\nonumber \\ \end{aligned}$$

where $\omega = \min (1/4, \delta /c_1^{1/\alpha }).$ By using that the equation (5.3) is time-reversible, we obtain together with (5.4) that

5.5 $$\begin{aligned} |u(x,1)| + |u_0(x)| \le K(u,V) |x| e^{-\tilde{\omega } |x|^{1/\alpha }}, \end{aligned}$$

where we let $\tilde{\omega } = \min \{ \omega , \delta /c_2^{1/\alpha } \},$ and we define the constant *K*(*u*, *V*) to be

$$\begin{aligned} C \left( 1 + \Vert (1+|z|)u_0\Vert _1 + \Vert (1+|z|)u(1)\Vert _1 +\Vert u\Vert _{L^{\infty }_{[0,1]} L^2(\mathbb {R})} \Vert V\Vert _{L^{\infty }_{[0,1]} L^2((1+|z|)^2 dz)} \right) . \end{aligned}$$

We now simply invoke the result by Escauriaza–Kenig–Ponce–Vega [5], even in a weak form: if *V* satisfies the conditions of Theorem 1 and $u_0, u(1) \in L^2(e^{A|x|^2},\mathbb {R})$ for $A \gg 1$ sufficiently large, then $u \equiv 0.$ By (5.5), we see that this condition is fulfilled as $\alpha < \frac{1}{2}$, and thus we have finished the proof of the Theorem. $\square$

We are now able to use Theorem 1 in order to conclude Corollary 1 about unique continuation for solutions to Schrödinger equations.

#### Proof of Corollary 1

If $u,v \in C([0,T] :H^s(\mathbb {R}) \cap L^2(x^2 \, dx))$ are solutions to (2.3), then $u-v =: {\tilde{w}}$ satisfies (2.1) for the potential

$$\begin{aligned} V = \frac{F(u,{\overline{u}}) - F(v,{\overline{v}})}{u-v}. \end{aligned}$$

If $F \in C^1(\mathbb {C}^2 :\mathbb {C}),$ one may find by (2.4) that

5.6 $$\begin{aligned} |V(x,t)| \le C(|u(x,t)|^{p-1} + |v(x,t)|^{p-1}). \end{aligned}$$

As $u,v \in L^{\infty }([0,T] :L^{\infty }(\mathbb {R})),$ the conditions on *u*, *v* imply that $V \in L^1([0,T] :L^{\infty }(\mathbb {R})) \cap L^{\infty }([0,T] :L^2((1+|x|)^2 dx)).$ By (5.6), *V* is seen to satisfy the other conditions in Theorem 1, and thus we may apply that result directly, which implies that $w \equiv 0,$ or equivalently $v \equiv u.$ $\square$

### Proof of the One-dimensional Rigidity Result

#### Proof of Corollary 2

We start off by noticing that from Lemma 4.1 from $k=1$, the solution $u \in C([0,T] :H^1(\mathbb {R})).$ From Lemma 4.7, we have that

$$\begin{aligned} |u_0(x)| + |u(x,T)| \lesssim _N e^{-N|x|}, \, \forall N \ge 1. \end{aligned}$$

We then use the following result by Kenig–Ponce–Vega:

#### Lemma 5.1

([14, Lemma 2.1]) For any $T>0$, there is $\varepsilon (T)>0$ so that the following holds: if $\lambda \in \mathbb {R}^d$ is a vector, $V:\mathbb {R}^d \times [0,T]\rightarrow {\mathbb {C}}$ is a potential so that

$$\begin{aligned} \Vert V\Vert _{L^1([0,T];L^{\infty }(\mathbb {R}^d))} \le \varepsilon , \end{aligned}$$

and $w \in C([0,T] :L^2(\mathbb {R}^d))$ is a solution to

$$\begin{aligned} i\partial _t w =- \Delta w + V\cdot w + F, \end{aligned}$$

then

$$\begin{aligned} \sup _{t \in [0,T]} \Vert e^{\lambda \cdot x} w(t)\Vert _{L^2(\mathbb {R}^d)}\lesssim & {} \Vert e^{\lambda \cdot x} w_0\Vert _{L^2(\mathbb {R}^d)} + \Vert e^{\lambda \cdot x} w(T)\Vert _{L^2(\mathbb {R}^d)} \\&+ \Vert e^{\lambda \cdot x} F(x,t)\Vert _{L^1([0,T]; L^2(\mathbb {R}^d))}. \end{aligned}$$

In our case, we shall apply this result with $V = \chi _{\mathbb {R}^d \setminus B_R} |u|^2$ and $F = \chi _{B_R} |u|^2 u.$ We clearly see that *V* satisfies the conditions of Lemma 5.1 if we pick $R >0$ sufficiently large. Thus, by applying this result with both $N,-N$ with the estimate above, we have

$$\begin{aligned} \sup _{t\in [0,T]} \Vert e^{N|x|} u(t) \Vert _2 \le C (\, \Vert e^{N|x|} u_0 \Vert _2 + \Vert e^{N|x|} u(T)\Vert _2 \, ) + e^{NR} \Vert u\Vert _{L^2_T L^{\infty }_x}^2 \Vert u\Vert _{L^{\infty }_T L^2_x}. \end{aligned}$$

In particular, we have that $e^{N|\cdot |} u(\cdot ,t) \in L^2$ for all $t \in [0,T].$ Now, we use Lemma 4.4 as well as (4.18) in order to conclude that there is a time

$$\begin{aligned}T' = T'(\Vert e^{N|x|} u_0\Vert _2,\Vert e^{N|x|}u(T)\Vert _2, \Vert u_0\Vert _{H^1}, \Vert u(T)\Vert _{H^1})\end{aligned}$$

so that for all *t*, $e^{\frac{ix^2}{4 \tau }} u(t+\tau )$ admits an analytic continuation in a strip of width $N \tau ,$ where $\tau < T'.$

In particular, if we take $t = T - \frac{T'}{2}, \tau = \frac{T'}{2}$ we will obtain that $e^{\frac{ix^2/4}{T'/2}} u(T)$ is analytic and bounded in a strip $S(r), r < \frac{N T'}{2}.$

In order to conclude, we need only to invoke Lemma 4.6. In fact, for $r < \frac{ N T'}{2}$ as above, we know that (4.20) holds in particular for all real zeros of $e^{\frac{ix^2/4}{T'/2}} u(T)$. But then we should have

5.7 $$\begin{aligned} \sum _{n \ge 0} \frac{1}{e^{c_2 \log (1+n)^{\alpha }/(2r)} - e^{-c_2 \log (1+n)^{\alpha }/(2r)}} < + \infty . \end{aligned}$$

But, in case $\alpha < 1,$ we have that $e^{c_2 \log (1+n)^{\alpha }/(2r)} < e^{\log (1+n)} = n+1$ for all $n \gg _{a,c_2} 1.$ Thus, we should have, by (5.7),

$$\begin{aligned} \sum _{n \ge 0} \frac{1}{n+1} < + \infty . \end{aligned}$$

This is a clear contradiction. This contradiction arises from the fact that, in order to use Lemma 4.6, we must assume $u(T) \not \equiv 0.$ Thus, $u(T) \equiv 0,$ and it follows at once that $u_0 \equiv 0,$ as desired. $\square$

## Proof of the Higher-dimensional Results

In this section, we will discuss how to prove a suitable version of the theorem above in higher dimensions.

### Proof of Theorem 3

We note that for $d=2,3$, we first use Lemma 4.2 to guarantee that $u_0$ and *u*(*T*) are in $H^1(\mathbb {R}^d)$. (For $d\ge 4$, this is already guaranteed as $s>s_c = d/2 -1$.) Now, we employ Lemma 4.9 for $N = 0$ in order to conclude the exponential decay of $u_0$ and *u*(*T*). From that, we use Lemma 4.2, which guarantees that $u_0, u(T) \in H^k(\mathbb {R}^d),$ for all $k \in \mathbb {N}.$

The next step is to employ Lemma 4.5, together with a Corollary of the argument of Kenig–Ponce–Vega from Lemma 5.1. Indeed, we use an induction argument to prove that, for each $N>0,$ there is $c_N > 0$ so that $e^{c_N|x|} \partial ^{\alpha }_x u(t) \in L^2(\mathbb {R}^d),$ for all $t\in [0,T]$ and for all $\alpha$ multiindices so that $|\alpha | \le N.$

Indeed, for $N=0,$ the result follows in the same fashion as the argument in the proof of Theorem 2. Assuming it holds for some $N \in \mathbb {N},$ a routine computation shows that any derivative $\partial _x^{\alpha } u =:v_{\alpha },$ $|\alpha | = N+1,$ satisfies an equation of the form

$$\begin{aligned} i \partial _t v_{\alpha } = - \Delta v_{\alpha } + |u|^2 v_{\alpha } + H, \end{aligned}$$

for *H* a finite sum of products of derivatives of *u* and ${\overline{u}}.$ The key property of *H* is that each of the summands is a product of some *lower order* derivative of *u* with two other derivatives of *u* of order $< N +1.$ By picking ${\tilde{V}} = \chi _{\mathbb {R}^d \setminus B_R} |u|^2$ and $F = H + \chi _{B_R} |u|^2,$ we see immediately that the conditions of Lemma 5.1 hold for some $R > 0$ sufficiently large. As we have proved in Lemma 4.9 that $\partial ^{\alpha }_x u_0, \partial _x^{\alpha } u(T) \in L^2(e^{c_{N+1}|x|})$ for some $c_{N+1} > 0,$ and the induction hypothesis shows that $H \in L^1([0,T]; L^2(e^{c_{N}|x|})),$ we conclude by Lemma Lemma 5.1 that $\sup _{t \in [0,T]} \Vert e^{c_{N+1}|x|}\partial ^{\alpha }_x u(t) \Vert _2 < + \infty ,$ as desired.

We are now in a position to use Lemma 4.5. Indeed, choosing $N = \lceil d/2 \rceil$ in the argument above, let $C(u) = \sup _{t \in [0,T]} \Vert u(t)\Vert _{{\mathcal {A}}^N_2(c_{N+1}/2)} < +\infty$ for shortness. Fix the time $T'(C(u))$ given by Lemma 4.5. We pick $t = T - \frac{T'(C(u))}{2},$ and from Lemma 4.5 itself and the comment thereafter we have that $e^{i|z|^2/(2T'(C(u)))}u(T) =: v(T)$ is analytic and bounded in each strip $S^d(r), \, r < \pi c_{N+1} T'(C(u)).$

In order to finish, we shall resort back to one-dimensional models. Indeed, if *v*(*T*) is as before, then we consider the one-dimensional functions $v(T)_{{\tilde{z}}}(z_1) = v(T)(z),$ where $z_1$ is the first coordinate of *z*,  and ${\tilde{z}}$ denotes the projection of the remaining coordinates onto $\mathbb {C}^{d-1}.$ We fix any ${\tilde{z}} \in \mathbb {R}^d$ for the rest of the argument.

By construction, $v(T)_{{\tilde{z}}}$ is bounded and analytic in any one (complex) dimensional strip $S^1(r')$ as above. But, by construction once more, we have that $v(T)_{{\tilde{z}}}$ has, when restricted to $z_i \in \mathbb {R},$ zeros of the form $(c_2^2 \log (n+1)^{2\alpha } - |{\tilde{z}}|^2)^{1/2},$ with $n > 0$ sufficiently large. But we may employ the same argument with Lemma 4.6 to this sequence as well, as it grows like $c_2 \log (1+n)^{\alpha }.$ This shows that $v(T)_{{\tilde{z}}}$ vanishes identically, and so does $u(T)_{{\tilde{z}}}.$ As ${\tilde{z}}$ was arbitrary, we conclude that $u(T) \equiv 0,$ as desired. $\square$
